# SETD1A modulates cell cycle progression through a miRNA network that regulates p53 target genes

**DOI:** 10.1038/ncomms9257

**Published:** 2015-09-23

**Authors:** Ken Tajima, Toshifumi Yae, Sarah Javaid, Oliver Tam, Valentine Comaills, Robert Morris, Ben S. Wittner, Mingzhu Liu, Amanda Engstrom, Fumiyuki Takahashi, Joshua C. Black, Sridhar Ramaswamy, Toshihiro Shioda, Molly Hammell, Daniel A. Haber, Johnathan R. Whetstine, Shyamala Maheswaran

**Affiliations:** 1 Massachusetts General Hospital Cancer Center, Harvard Medical School, Charlestown, Massachusetts 02129, USA; 2 Cold Spring Harbor Laboratory, Cold Spring Harbor, New York 11724, USA; 3 Department of Surgery, Harvard Medical School, Charlestown, Massachusetts 02129, USA

## Abstract

Expression of the p53-inducible antiproliferative gene *BTG2* is suppressed in many cancers in the absence of inactivating gene mutations, suggesting alternative mechanisms of silencing. Using a shRNA screen targeting 43 histone lysine methyltransferases (KMTs), we show that SETD1A suppresses BTG2 expression through its induction of several BTG2-targeting miRNAs. This indirect but highly specific mechanism, by which a chromatin regulator that mediates transcriptional activating marks can lead to the downregulation of a critical effector gene, is shared with multiple genes in the p53 pathway. Through such miRNA-dependent effects, SETD1A regulates cell cycle progression *in vitro* and modulates tumorigenesis in mouse xenograft models. Together, these observations help explain the remarkably specific genetic consequences associated with alterations in generic chromatin modulators in cancer.

## Abstract (web summary)

The p53-inducible antiproliferative gene *BTG2* is suppressed in many cancers, in the absence of inactivating gene mutations. Here the authors show that the histone lysine methyltransferase SETD1A suppresses the expression of several p53 target genes including *BTG2* by inducing a network of microRNAs.

## Graphical abstract

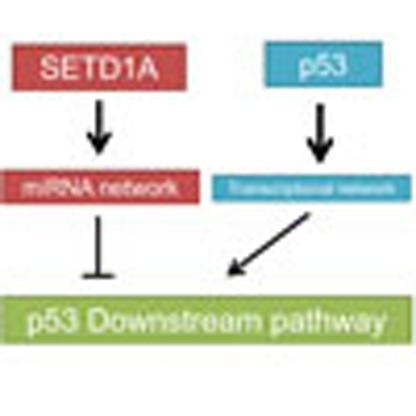

B*TG2*, a p53-inducible gene, plays a critical role in p53-mediated suppression of mouse and human fibroblast transformation induced by oncogenic-Ras^1^. BTG2 inhibits cell cycle progression and its depletion enhances tumorigenesis in mouse models. In human cancers, including breast and prostate cancers, BTG2 expression is suppressed^2^. Despite its tumour-suppressor effects, mutations in BTG2 have not been detected, suggesting that epigenetic mechanisms could be involved in modulating BTG2 expression in cells.

Chromatin-modifying enzymes are emerging as important regulators of tumorigenesis, as well as promising cancer therapeutic targets. Despite their broad effects on chromatin structure, they also have striking target specificity, a paradox that is poorly understood. Histone methylation, catalysed by lysine methyltransferases (KMT), has been linked to both transcriptional activation (H3K4, H3K36 and H3K79) and repression (H3K9, H3K27 and H4K20)^3^. The Set1/COMPASS family of H3K4 methyltransferases, first identified in *Saccharomyces cerevisiae*, consists of several members in humans and is associated with transcriptional activation^4^. Although H3K4 methylation is associated with transcriptional activation, the overall change in gene expression landscape within a cell may depend on complex interplay between the target genes induced, which may lead to secondary effects that repress as well as induce gene expression.

Here we conducted an unbiased short hairpin RNA (shRNA) screen against 43 KMTs to determine whether baseline expression of BTG2 in proliferating cells is regulated by epigenetic mechanisms. We identified SETD1A as a KMT whose knockdown strongly induces activation of BTG2. This paradoxical repressive effect of an activating chromatin regulator results from its transcriptional activation of multiple microRNAs (miRNAs), which themselves suppress the BTG2 transcript. This effect is consistent with the roles of miRNAs in coordinating multiple downstream targets that play important roles in cellular differentiation, proliferation and signalling^5^,^6^. In addition to targeting BTG2, SETD1A-induced miRNAs target additional downstream effectors of p53, as well as cell cycle regulators. These findings illustrate the exquisite target specificity exhibited by chromatin-modifying enzymes, and the complex network through which chromatin regulators influence genes involved in tumorigenesis.

## Results

### Identification of SETD1A as a regulator of BTG2 expression

**Figure 1:**
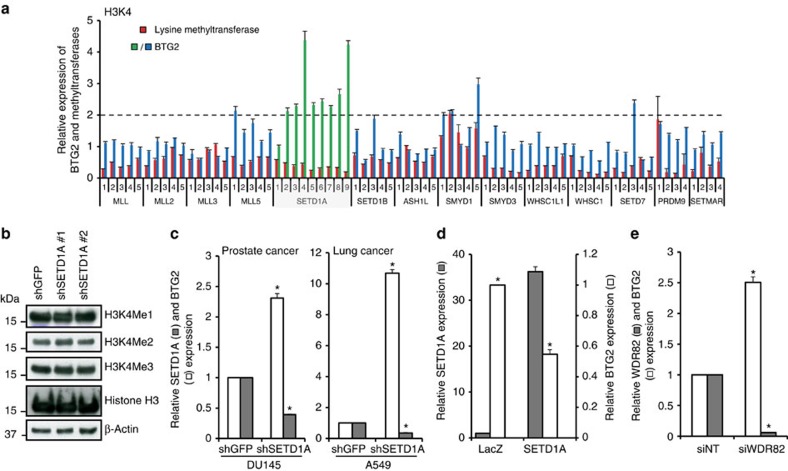
BTG2 expression is regulated by SETD1A. (**a**) Knockdown of SETD1A specifically induces BTG2. A lentiviral shRNA screen against H3K4 KMTs in MDA-MB-231 cells shows that SETD1A depletion specifically induces BTG2 expression compared with pLKO-infected control cells. Fold change in BTG2 and KMT expression after 72 h of viral infection is shown with controls set at 1. The dotted line marks twofold induction of BTG2 compared with control. KMTs and BTG2 are shown as red and blue/green bars, respectively. (**b**) Total proteins isolated from control and SETD1A-depleted MDA-MB-231 cells were analysed for H3K4Me1, H3K4Me2, H3K4Me3, total histone H3 and ß-actin protein expression. (**c**) SETD1A suppresses BTG2 in prostate and lung cancer cells. SETD1A and BTG2 expression in SETD1A-depleted cells represents the average derived from cells individually infected with the two different shSETD1A constructs (shSETD1A#1 and shSETD1A#2), with the shGFP-infected control set at 1. (**d**) Expression of lentivirally expressed SETD1A in MCF10A cells decreases BTG2 expression (white bar). Fold expression of SETD1A in control and SETD1A-infected cells is shown (black bar). (**e**) WDR82, a non-catalytic subunit of the SET1/COMPASS complex, regulates BTG2 expression. WDR82 and BTG2 expression in WDR82-depleted MDA-MB-231 cells represents the average derived from cells individually transfected with the two different siWDR82 sequences, with the siNT (non-targeting, NT)-transfected control set at 1. Data are represented as mean±s.d. of the average of three experimental replicates. Asterisks indicate *P* values of *P*<0.05 for **a**,**c**–**e** by Student's *t*-test.

To investigate whether epigenetic silencing is involved in suppressing BTG2 expression in proliferating cells, we measured BTG2 induction in the human breast cancer cell line MDA-MB-231, following infection with an arrayed lentiviral shRNA library containing shRNAs against 43 KMTs. Four to ten independent shRNA constructs were tested, targeting each KMT implicated in chromatin marks, H3K4, H3K9, H3K27, H3K36, H3K79 and H4K20 methylation, as well as unknown substrates (Supplementary Fig. 1a). Baseline BTG2 mRNA is undetectable in MDA-MB-231 cells, facilitating measurement of its induction 3 days after infection. Successful knockdown of KMT expression (>40%) with at least two shRNAs, compared with control pLKO vector-infected cells, was observed for 32 of the 43 targeted KMTs (Fig. 1a and Supplementary Fig. 1b). Among these, knockdown of SETD1A by eight of nine shRNAs was unique in leading to a 2–4.5-fold increase in BTG2 mRNA (Fig. 1a). Knockdown of no other KMT led to a significant and robust increase or decrease in BTG2 expression (Fig. 1a and Supplementary Fig. 1b). Depletion of SETD1A, a KMT whose expression is increased in breast, prostate and lung cancers^7^,^8^ (Supplementary Fig. 2a), did not alter total H3K4Me1, H3K4Me2 and H3K4Me3 and histone H3 levels in cells (Fig. 1b and Supplementary Fig. 8a). BTG2 mRNA induction (up to 10-fold) on SETD1A depletion was observed in multiple breast (*n*=4), prostate (*n*=2), lung (*n*=2) and colon (*n*=6) cancer cell lines, and it was independent of the mutational status of p53, a known regulator of BTG2 (Fig. 1c and Supplementary Fig. 2b; *P*<0.05, Student's *t*-test). In addition to mRNA quantification, SETD1A-mediated BTG2 regulation was validated using western blot analysis (Supplementary Fig. 2c and Supplementary Fig. 8b). Further, ectopic overexpression of SETD1A in MCF10A breast epithelial cells, which express BTG2 at baseline, led to reduced expression of the endogenous transcript (Fig. 1d; *P*<0.05, Student's *t*-test). Depletion of WDR82, a non-catalytic subunit specific to the SET1/COMPASS complexes (SETD1A and SETD1B) and not shared by the MLL/COMPASS complexes^4^, also induced BTG2 expression (Fig. 1e). Consistent with these findings, analysis of a large data set consisting prostate tumours^9^,^10^ showed a significant inverse correlation between BTG2 and SETD1A mRNA expression in tumours, an effect that was most pronounced in lower-grade prostate tumours (Gleason score ≤6). No such correlation was present in normal prostate tissue (Supplementary Fig. 3a); a similar negative correlation was also observed in breast^11^ and lung^12^,^13^ cancers (Supplementary Fig. 3b).

### SETD1A suppresses BTG2 expression through a miRNA network

Having identified a pronounced effect of a KMT chromatin regulator on BTG2 expression, we first analysed the chromatin marks at the BTG2 promoter. Tiled chromatin immunoprecipitation–quantitative PCR (ChIP–qPCR) across a 5-kb region spanning the BTG2 transcriptional start site (Supplementary Fig. 4a) demonstrated peaks of H3K4Me1, H3K4Me2 and H3K4Me3 marks in the proximity of the transcription start site (Supplementary Fig. 4a–d). SETD1A depletion led to a significant decline in the H3K4Me3 peaks as well as SETD1A occupancy within this area (Supplementary Fig. 4d,e; *P*<0.05, Student's *t*-test) suggesting that SETD1A maintains the activating H3K4 methylation marks on the BTG2 promoter. As control, depleting MLL did not affect H3K4 marks or MLL occupancy on the BTG2 promoter, suggesting that BTG2 is a specific target of SETD1A (Supplementary Fig. 4f,g). However, the presence of activating chromatin marks on the BTG2 promoter would have suggested that SETD1A depletion should result in decreased BTG2 expression, rather than the observed BTG2 induction. To better understand this apparent paradox, we investigated whether SETD1A might also induce negative regulators of BTG2, namely miRNAs.

**Figure 2:**
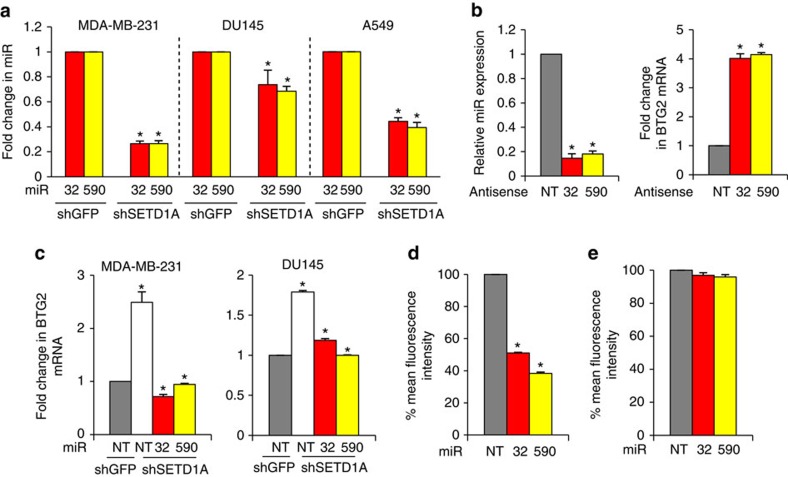
SETD1A regulates BTG2 through induction of miRNAs. (**a**) Regulation of BTG2 expression by SETD1A is mediated through miRNAs. SETD1A regulates miRNA-32 and -590-5p, whose expression was analysed using qPCR in shGFP and shSETD1A-infected MDA-MB-231, DU145 and A549 cells. Expression of miRNAs in controls was set at 1. Expression of the miRNAs in SETD1A-depleted cells represents the average derived from cells individually infected with two shSETD1A constructs. (**b**) Suppression of miRNA-32 and -590-5p with Anti-miRs induces BTG2 expression. Expression of the two miRNAs indicated was suppressed with Anti-miRs, and each miRNA (left) and BTG2 mRNA (right) was measured using qPCR. The level of each miRNA and BTG2 in cells transfected with non-targeting (NT) sequences was set at 1. (**c**) Expression of miRNA-32 and -590-5p abrogates BTG2 induction in SETD1A-depleted cells. BTG2 expression in shGFP-infected control cells transfected with NT sequences was set at 1. (**d**) miRNA-32 and -590-5p, target the 3′-UTR of BTG2. A plasmid containing GFP linked to 3′-UTR of BTG2 was co-transfected with the two miRNA sequences into 293T cells, and the intensity of GFP expression was monitored by fluorescence-activated cell sorting. NT sequences were used as control and the intensity of GFP in this sample was set at 100%. (**e**) Deletion of the region harbouring binding sites for miRNA-32 and -590 from the 3′-UTR of BTG2 abrogates miRNA-32- and -590-5p-mediated suppression of the 3′-UTR of BTG2. Data are represented as mean±s.d. of the average of three experimental replicates. Asterisks indicate *P* values of *P*<0.05 for all figures by Student's *t*-test. For the relevant figures, NT represents NT, and 590 indicates miRNA-590-5p.

We screened for miRNAs differentially expressed in shSETD1A or shGFP-infected MDA-MB-231 cells. SETD1A knockdown using two independent shRNAs led to an upregulation of 67 miRNAs (more than twofold) and suppression of 80 miRNAs (∼60%; Supplementary Table 1). Three target prediction algorithms, TargetScan^14^, miRanda^15^ and PicTar^16^, identified the downregulation of five BTG2-targeting miRNAs, miR-590-5p, miR-27a, miR-186, miR-20b and miR-32, in SETD1A-depleted cells. We further validated and characterized two BTG2-targeting miRNAs: miR-590-5p, which shows the highest levels of suppression (∼75%) in SETD1A-depleted cells, and the previously identified BTG2-targeting miR-32 (ref. 17). Depletion of SETD1A suppressed both miRNAs in multiple cell lines (Fig. 2a and Supplementary Fig. 5a; *P*<0.05, Student's *t*-test). Suppressing the miRNAs with corresponding anti-miRs increased BTG2 mRNA two- to fourfold (Fig. 2b and Supplementary Fig. 5b; *P*<0.05, Student's *t*-test), whereas restoration of either miRNAs individually into SETD1A-depleted cell lines abolished BTG2 induction (Fig. 2c and Supplementary Fig. 5c; *P*<0.05, Student's *t*-test). Expression of a green fluorescent protein (GFP) construct linked to the 3′-untranslated repeat (UTR) of BTG2 demonstrated that both miRNAs suppress GFP expression in cells (Fig. 2d). Deletion of the region harbouring the two miRNA-32-binding sites and the single miRNA-590-binding site from the 3′-UTR of BTG2 abrogated this effect (Fig. 2e and Supplementary Fig. 5d). Thus, the repression of miRNA-32 and -590-5p expression by SETD1A depletion may contribute to the increase in BTG2 mRNA levels.

Interestingly, expression of both miR-32 and -590-5p is increased in breast cancer samples compared with normal tissue (*P*=1.4*e*^−31^ for miR-32, *P*=6.18*e*^−09^ for miR-590-5p, Student's *t*-test; Supplementary Fig. 5e), and an inverse correlation is observed between BTG2 expression and miRNA-32 and -590-5p, supporting the relevance of these findings in clinical specimens (cor=−0.244, *P*=1.33*e*^−11^ for BTG2/miR-32; cor=−0.461, *P*=1.32*e*^−40^ for BTG2/miR-590-5p, Pearson's correlation; Supplementary Fig. 5f). Together, these results suggest that SETD1A-induced miRNAs suppress BTG2 expression.

### SETD1A regulates the promoters of BTG2-targeting miRNAs

**Figure 3:**
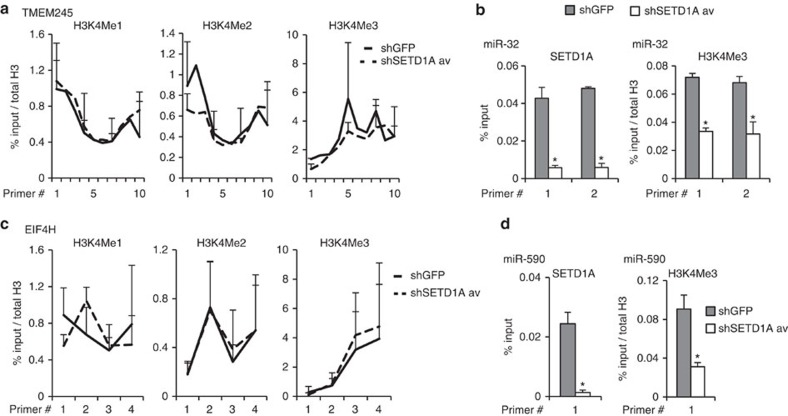
SETD1A regulates the promoters of miR-32 and miR-590-5p. (**a**) H3K4Me1, H3K4Me2 and H3K4Me3 marks on the promoter region of TMEM245 were analysed using 10 primers that span the region. The results show that SETD1A does not regulate these chromatin marks on the TMEM245 promoter. (**b**) SETD1A binding and H3K4Me3 on the putative promoter region of miR-32 were analysed using two primers that span the region. The results show that SETD1A depletion suppresses H3K4 methylation and SETD1A binding to this domain. (**c**) H3K4Me1, H3K4Me2 and H3K4Me3 on the promoter region of EIF4H were analysed using four primers that span the region. The results show that SETD1A does not regulate these chromatin marks on the EIF4H promoter. (**d**) SETD1A binding and H3K4Me3 marks on the putative promoter region of miR-590 was analysed using a single primer that spans the region. The results show that SETD1A depletion suppresses H3K4 methylation and SETD1A binding to this domain. For all experiments, shSETD1A data points represent the average derived from ChIP assays performed with cells individually infected with two different shSETD1A constructs. Data are represented as mean±s.d. of the average of three experimental replicates. Asterisks indicate *P* values of *P*<0.05 for relevant figures by Student's *t*-test.

We then determined whether SETD1A directly regulates the expression of BTG2-targeting miRNAs. miR-32 resides within intron 12 of the host gene *TMEM245*, whose promoter is ∼90 kb away from the miRNA (Supplementary Fig. 6a, green oval). H3K4Me1, H3K4Me2 and H3K4Me3 levels of the TMEM245 promoter do not change on SETD1A knockdown (Fig. 3a). Thus, the suppression of miR-32 is not due to TMEM245 host gene regulation. The UCSC genome database revealed a proximal H3K4Me3 peak ∼4 kb away from miR-32 (Supplementary Fig. 6a, red oval). ChIP analysis of this region demonstrates that both SETD1A binding and H3K4Me3 are significantly reduced on SETD1A depletion (Fig. 3b; *P*<0.05, Student's *t*-test).

We similarly evaluated miR-590, which localizes within the host gene *EIF4H*, whose promoter is ∼15 kb upstream of the miRNA (Supplementary Fig. 6b, green oval). Again, H3K4 methylation of the EIF4H promoter does not change on SETD1A knockdown (Fig. 3c). Analysis of the region coinciding with the miR-590 promoter previously described^18^ (Supplementary Fig. 6b, red oval) demonstrates a significant decrease in both SETD1A binding and H3K4Me3 on SETD1A knockdown (Fig. 3d; *P*<0.05, Student's *t*-test). These results reveal that BTG2-targeting miRNAs, miR-32 and miR-590, are both direct targets of SETD1A and that they are induced independently of their host transcripts by this chromatin regulator.

### SETD1A-induced miRNAs are enriched for p53 target genes

**Figure 4:**
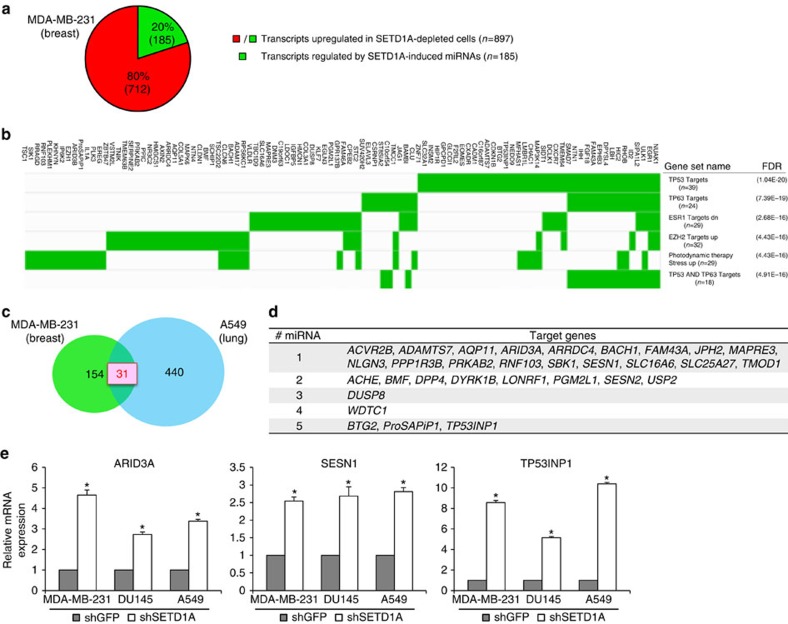
SETD1A-induced miRNAs are enriched for regulators of the p53 downstream pathway and cell cycle-regulatory genes. (**a**) Schematic representation of the genes upregulated in SETD1A-depleted MDA-MB-231 cells. Twenty per cent of the genes (185 out of 897 mRNAs) upregulated in SETD1A-depleted cells are high-confidence targets (green) of SETD1A-regulated miRNAs. (**b**) The six top categories of enriched gene signatures in the GSEA database are shown for the 185 genes regulated by SETD1A-induced miRNAs. Genes contributing to the enrichment are highlighted in green. The number of genes contributing to the enriched signatures within each category is given in parentheses. FDR was determined with *P* value by the gene set enrichment hypergeometric test. (**c**) Schematic representation of SETD1A-induced miRNA target genes that are common to both MDA-MB-231 and A549 cells. Comparison of the 185 target genes regulated by SETD1A-induced miRNAs against 471 genes induced in SETD1A-depleted A549 cells identified 31 common genes. (**d**) List of the 31 common genes targeted by SETD1A-induced miRNAs. The number of miRNAs regulating each gene is listed on the left. (**e**) Expression of p53 downstream target genes shown were analysed using qPCR in shGFP- and shSETD1A-infected MDA-MB-231, DU145 and A549 cells. Expression in SETD1A-depleted cells represents the average derived from cells individually infected with the two shSETD1A constructs, with the shGFP-infected control set at 1. Data are represented as mean±s.d. of the average of three experimental replicates. Asterisks indicate *P* values of *P*<0.05 by Student's *t*-test.

The effect of SETD1A on BTG2 expression may illustrate a broader miRNA network by which this chromatin regulator modulates genes involved in tumorigenesis. To evaluate the full effect of SETD1A-induced miRNA targets, we identified microRNA–mRNA interactions among all genes and miRNAs differentially expressed in SETD1A-depleted MDA-MB-231 cells using TargetScan, miRanda and PicTar^14^,^15^,^16^. Only those identified by all three algorithms were selected to form a high-confidence set of consensus microRNA targets. Of the 897 genes induced in SETD1A knockdown MDA-MB-231 cells, only 185 (20%) genes were miRNA targets (Fig. 4a and Supplementary Table 2). Gene set enrichment analysis (GSEA) of these 185 genes identified 100 gene signatures (*P*<7.06*e*^−07^, false discovery rate (FDR) *q*-value (FDR) <4.50*e*^−05^, hypergeometric test; Fig. 4b and Supplementary Table 3), with enrichment for genes induced by p53 being the top hit (*P*=1.6*e*^−24^, FDR=1.04*e*^−20^, hypergeometric test^19^). Additional pathways significantly enriched were TP63 (*P*=2.32*e*^−22^, FDR=7.39*e*^−19^, hypergeometric test^19^) and ESR1 (*P*=1.26*e*^−19^, FDR=2.68*e*^−16^, hypergeometric test^20^) targets (Fig. 4b and Supplementary Table 3).

Comparison of these 185 miRNA targets induced in SETD1A-depleted MDA-MB-231 cells against the set of genes comparably induced in SETD1A-depleted A549 cells identified 31 consensus genes (Fig. 4c,d). The 31 common SETD1A-regulated genes, thus identified, were each found to be targeted by —one to five SETD1A-induced miRNAs (Fig. 4d and Supplementary Table 4). *BTG2* is one of three genes (*BTG2*, *ProSAPiP1* and *TP53INP1*) targeted by the highest number of SETD1A-regulated miRNAs (Supplementary Table 4). GSEA of the 31 genes also showed significant enrichment for canonical p53 downstream pathway genes (*P*=2.23*e*^−6^, FDR=2.95*e*^−3^, Benjamini–Hochberg method). Among the downstream components most significantly enriched, and independently confirmed in multiple cell lines using qPCR, are ARID3A, SESN1 and TP53INP1 (Fig. 4e). Additional computational analyses (Gene, Disease Features Ontology-based Overview System) showed that 21 of the 31 genes common between MDA-MB-231 and A549 cells (targeted by 24 SETD1A-induced miRNAs) were significantly related to the p53 pathway, neoplasms and cell cycle regulation (Supplementary Fig. 7a); StarBase enrichment analysis^21^,^22^ of these 24 SETD1A-induced miRNAs shows that they are significantly enriched for components associated with negative regulation of cell proliferation, and p53 and cancer-associated pathways (Supplementary Fig. 7b). Taken all together, these results show that SETD1A-regulated miRNAs target multiple genes involved in cell cycle regulation, and p53 downstream pathways, including BTG2.

### SETD1A depletion inhibits the cell cycle and tumour growth

**Figure 5:**
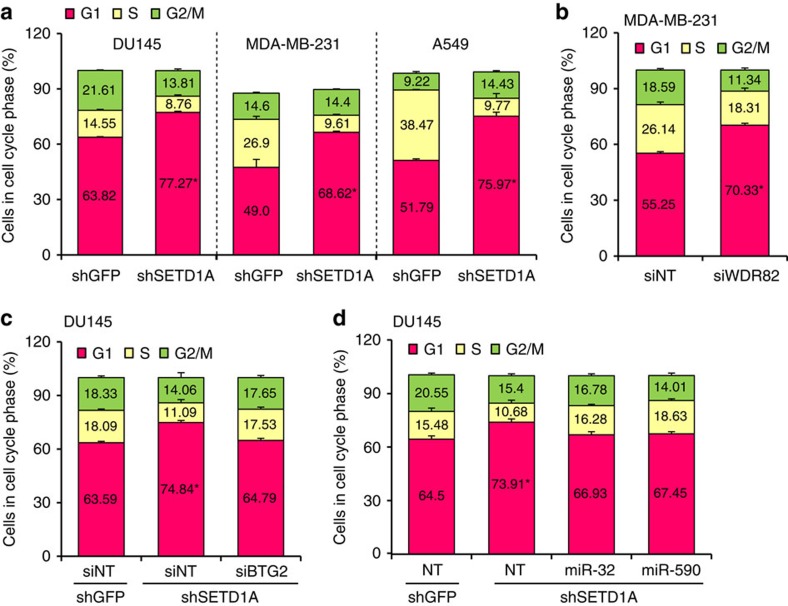
SETD1A regulates cell cycle progression. (**a**) SETD1A depletion increases the G1 fraction. Cell cycle analysis of MDA-MB-231, A549 and DU145 cells infected with shGFP and shSETD1A constructs. The per cent cell cycle distribution of SETD1A-depleted cells represents the average derived from cells individually infected with the two different shSETD1A constructs. (**b**) WDR82 depletion increases the G1 fraction. Cell cycle analysis of MDA-MB-231 cells transfected with siWDR82 and siNT sequences. The per cent cell cycle distribution of WDR82-depleted cells represents the average derived from cells individually transfected with the two different siWDR82 constructs. (**c**) Depletion of BTG2 partially reverses the increase in the G1 fraction exhibited by SETD1A knockdown cells. Cell cycle analysis of shSETD1A-infected cells transfected with siBTG2 and NT siRNA (siNT). shGFP-infected cells transfected with siNT are shown as control. As previously seen, knockdown of SETD1A (siNT) increases the fraction of cells in G1 compared with shGFP (siNT)-infected cells. Suppression of BTG2 with siBTG2 in shSETD1A cells reverses this effect. (**d**) miRNAs reverse the G1 increase in SETD1A-depleted cells. Cell cycle analysis of shSETD1A-infected cells transfected with miRNA-32 and -590 mimics. Knockdown of SETD1A (siNT) increases the fraction of cells in G1 compared with shGFP (siNT)-infected cells. The mimics against miRNA-32 and -590 partially suppress this effect. Data are represented as mean±s.d. of the average of three experimental replicates. Asterisks indicate *P* values of *P*<0.001 for **a**,**b** and *P*<0.005 for **c**,**d** by Student's *t*-test.

**Figure 6:**
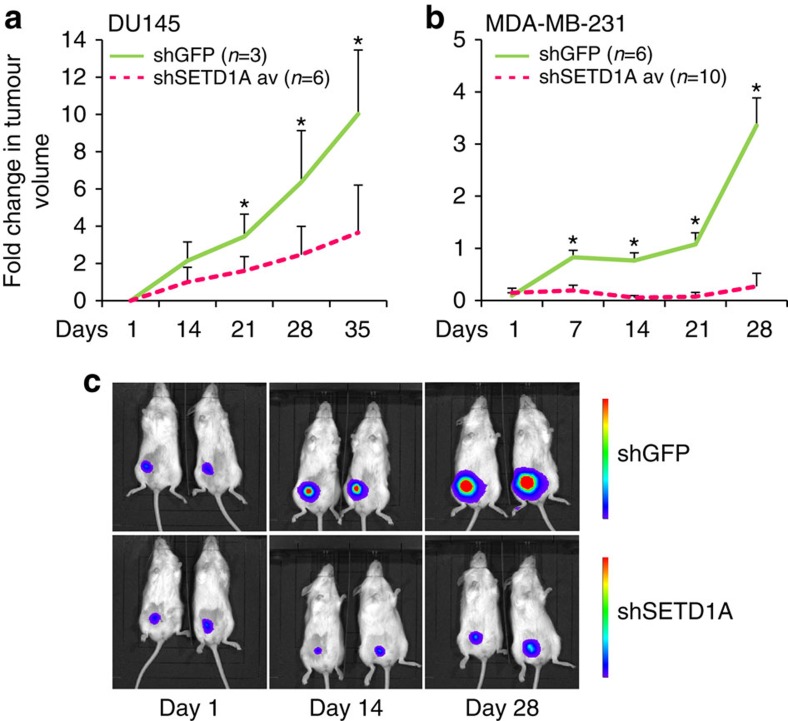
SETD1A regulates tumorigenesis. (**a**) DU145 cells infected with shGFP- and shSETD1A lentiviruses were inoculated subcutaneously into immunocompromised mice. Fold change in tumour volume and the number of animals in each group are shown. The volume of SETD1A-depleted tumours represents the average derived from cells individually infected with the two different shSETD1A constructs. Data are represented as mean±s.d. of the average of each group. Asterisks indicate *P* values of *P*<0.05 by Student's *t*-test. (**b**) Luciferase expressing MDA-MB-231 cells infected with shGFP- and shSETD1A lentiviruses were inoculated into the mammary fat pad of immunocompromised mice. Tumour growth was monitored by bioluminescence imaging. The fluorescence intensity of SETD1A-depleted tumours represents the average derived from tumour cells individually infected with the two different shSETD1A constructs. The number of animals in each group are shown. Data are represented as mean±s.d. of the average of each group. Asterisks indicate *P* values of *P*<0.05 by Student's *t*-test. (**c**) Serial bioluminescence imaging of shGFP and shSETD1A-MDA-MB-231 tumour-bearing mice from experiment described above (Fig. 6b) is shown.

To determine the functional consequence of SETD1A, we analysed cell cycle distribution in SETD1A-depleted cells. Consistent with the cell cycle-regulatory effects of BTG2 (ref. 2), depletion of SETD1A modulates cell cycle progression in cancer cells. In MDA-MB-231 as well as in A549 lung cancer cells and in DU145 prostate cancer cells, SETD1A depletion increased the G1 fraction (Fig. 5a; *P*<0.001, Student's *t*-test). A similar increase in the G1 fraction was also observed in WDR82-depleted MDA-MB-231 cells (Fig. 5b). The increase in G1 induced by SETD1A knockdown was partially reversed using siRNA-mediated inhibition of BTG2 (Fig. 5c; *P*<0.005, Student's *t*-test). Consistent with this effect, ectopic expression of miR-32 and -590-5p mimics reduced the G1 fraction in SETD1A knockdown cells (Fig. 5d; *P*<0.005, Student's *t*-test). The effect of SETD1A on cell proliferation was even more striking *in vivo*, where SETD1A knockdown abrogated tumorigenesis of DU145 and MDA-MB-231 cells (Fig. 6a–c; *P*<0.05, Student's *t*-test).

## Discussion

SETD1A-mediated regulation of BTG2 reveals a new dimension in the complex regulatory pathways mediated by chromatin-modifying enzymes. Although SETD1A regulates H3K4Me3 methylation of the BTG2 promoter, suggesting direct transcriptional regulation of BTG2, it concurrently induces the expression of several BTG2-targeting miRNAs. The ultimate consequence of these opposing signals is the suppression of BTG2 expression, reflecting the dominance of the miRNA-mediated effects. Analysis of SETD1A-regulated miRNA/mRNA targets across multiple cell lines shows that SETD1A-induced miRNA network broadly suppresses the expression of p53 downstream targets and cell cycle-regulatory genes in addition to BTG2. Of note, the suppression of p53 downstream targets, ARID3A, SESN1, TP53INP1 and BTG2, by SETD1A-induced miRNAs is independent of the cellular p53 status. These findings identify a subset of p53 target genes specifically induced by a chromatin modifier through a p53-independent regulatory mechanism, and illustrate the exquisite specificity and complexity involved in SETD1A-regulated gene expression.

Consistent with the induction of the p53 downstream targets, BTG2, TP53INP1 and SESN1, all of which have tumour-suppressor functions on their own^2^,^23^,^24^, SETD1A depletion suppresses tumour growth *in vivo*. This establishes a new functional role of SETD1A, which has previously been identified as developmentally required for gastrulation, but dispensable for mouse embryo implantation^25^. SETD1A is responsible for maintenance of the majority of H3K4 methylation in embryonic stem cells. Our data, however, demonstrate that SETD1A is not required for overall maintenance of H3K4 methylation in tumour cells (Fig. 1b), suggesting that SETD1A activity is dependent on the cellular context. Protein–protein interactions defining context-dependent H3K4 trimethylation by Set1/COMPASS has been reported in yeast^26^. Nonetheless, depletion of SETD1A in both tumour cells and embryonic stem cells interferes with cell cycle progression by increasing G1 (ref. 25). Whether SETD1A-mediated cell cycle regulation in embryonic stem cells involves miRNA intermediates remains to be determined.

In conclusion, the regulation of BTG2 by SETD1A serves as a model to illustrate (1) the exquisite target specificity exhibited by histone lysine methyltransferases, (2) a miRNA network-mediated mechanism through which SETD1A, a positive chromatin regulator of transcription, suppresses the expression of several genes involved in cell cycle regulation and the p53 pathway and (3) a new role for SETD1A in regulating tumour growth. Given that the misregulation of H3K4 methylation resulting from MLL fusion proteins, mutant MLL3 and MLL4 proteins, and aberrant demethylase activity has been implicated in cancer progression^27^,^28^,^29^, our observations linking the H3K4 regulator SETD1A in tumorigenesis supports the importance of this chromatin modifier and the potential application of small molecules targeting the enzymatic activity of KMTs in cancer.

## Methods

### Cell culture

Human breast, prostate and lung cancer cell lines MDA-MB-231, MDA-MB-468, MCF7, BT549, DU145, LNCaP, A549 and H1299 were grown in Dulbeccco's modified medium. Human colon cancer cell lines HCT8, HCT116, H630, DLD1, SW620 and HT29 were grown in RPMI 1640 medium. Both growth media were supplemented with 10% fetal bovine serum and penicillin/streptomycin (Pen/Strep). The non-tumorigenic MCF10A cells were cultured in DMEM/F12 medium with 5% horse serum, epidermal growth factor, Hydrocortizole, Cholera Toxin, Insulin and Pen/Strep. All cell lines were purchased from the American Type Culture Collection (ATCC, Manassas, VA, USA). All cell lines were maintained in 5% CO_2_ at 37 °C.

### Screening for KMTs that modulate BTG2 expression

Lentiviral shRNA constructs were obtained from the RNAi Consortium shRNA Library at the Broad Institute. Conditioned medium containing infective lentiviral particles was generated by co-transfecting 3 μg of lentiviral vector (plko1), 3 μg of pCMV d8.91 and 1 μg pHCMV-G into 1 × 10^6^ 293T human embryonic kidney cells using FuGENE 6 transfection reagent (Roche Applied Science). Supernatants were collected 48 h after transfection and were filtered through a 0.45-μM membrane (Millipore). Cells were infected using 8 μg ml^−1^ polybrene and selected with 2 μg ml^−1^ puromycin. RNA was isolated 72 h after infection and analysed using qPCR for expression of BTG2 and each histone lysine methyltransferase. The primer sequences used to analyse expression using qPCR are provided in Supplementary Table 6.

### Chromatin immunoprecipitation assay

SETD1A binding and H3K4 methylation of BTG2, TMEM245, EIF4H, miR-32 and -590 were analysed using ChIP. The genomic regions to be analysed were chosen on the basis of the H3K4Me3 profiles obtained from the UCSC data. Cells were grown to 80% confluence, crosslinked with 1% formaldehyde for 15 min at 37 °C and quenched with formaldehyde containing 125 mM glycine. After washing twice with cold PBS, cells were collected into 1 ml of lysis buffer (5 mM PIPES, 85 mM KCl and 0.5% NP-40, Protease Inhibitors). Nuclei were collected and incubated in nuclear lysis buffer (50 mM Tris (pH 8.0), 10 mM EDTA (pH 8.0), 0.2% EDTA, Protease Inhibitors). The crosslinked chromatin was sonicated into DNA fragments of ∼300–500 bp in length. Protein G beads were first incubated on a rotator at 4 °C for 6 h with 4 μg each of the following antibodies against Histone H3 (Abcam, ab1791), Histone H3 monomethyl K4 (Abcam, ab8895), Histone H3 dimethyl K4 (Abcam, ab11946), Histone H3 trimethyl K4 (Abcam, ab8580), SETD1A (BETHYL, A300–289A), MLL (BETHYL, A300–374A) and control IgG (Cell Signaling, 2729S). Chromatin solution (100 μg) was incubated with protein G magnetic beads conjugated with antibodies overnight on a rotator at 4 °C. After six washes, the beads were eluted with elution buffer (50 mM NaHCO_3_, 140 mM NaCl, 1% SDS). Following both RNaseA and proteinase K treatments, and reverse crosslinking, DNA was purified with the PCR clean-up kit (Qiagen) and analysed with qPCR using primers against the relevant targets listed in Supplementary Table 5.

### Western blot analysis

The antibodies used for western blot analysis were rabbit anti-SET1 polyclonal antibody (1:1,000, BETHYL, A300–289A), rabbit anti-BTG2 polyclonal antibody (1:2,000, GenWay Biotech, GWB-D54FE7), rabbit anti-Histone H3 antibody (1:1,000, Abcam, ab1791), rabbit anti-Histone H3 monomethyl K4 antibody (1:500, Abcam, ab8895), Goat anti-Histone H3 dimethyl K4 antibody (1:1,000, Abcam, ab11946), rabbit anti-Histone H3 trimethyl K4 antibody (1:1,000, Abcam, ab8580) and mouse anti-Actin monoclonal antibody (1:5,000, BD Bioscience, 612656). Cells were lysed in RIPA buffer (20 mM Tris, pH 8.0, 150 mM NaCl, 10 mM NaF, 0.1% SDS, 1% Nonidet P-40 and 1 × protease inhibitor mixture (Roche)), proteins were separated on a 4–15% polyacrylamide gradient–SDS gel (Bio-Rad), transferred on polyvinylidene difluoride membranes (Millipore) and blocked in TBST (Tris-buffered saline and Tween 20; 25 mM Tris, pH 7.4, 136 mM NaCl, 5 mM KCl and 0.1% Tween) containing 5% milk. The antibodies were used at each dilution, as described above, in 1% milk/TBST before use. Blots were incubated with the primary antibodies for 2 h at room temperature. Blots were washed (three times) with 1% milk/TBST and were incubated with the appropriate horseradish peroxidase-conjugated antibodies. Bound antibodies were detected with enhanced chemiluminescence (ECL) (Amersham Pharmacia Biotech).

### Validation of SETD1A-regulated miRNAs

Briefly, RNA isolated from shGFP- and shSETD1A (shSETD1A#1 and shSETD1A#2)-infected MDA-MB-231 cells, 3 days after infection, was assayed with an A & B card consisting of probes against a total of 756 miRNAs. The effect of SETD1A knockdown on miRNA expression was calculated as the ratio of miRNA expression in shSETD1A-MDA-MB-231 and shGFP-MDA-MB-231 cells. An arbitrary threshold of 60% suppression and twofold increase in expression were used to determine the miRNA population that was regulated by SETD1A.

For further evaluation of selected targets, total RNA including miRNA was isolated from cells using the mirVana miRNA Isolation Kit (Ambion). miRNAs were detected using a two-step qRT–PCR, with the first step being reverse transcription (RT) of cDNA and the second step being real-time qPCR. All reagents were supplied by Applied Biosystems. The RT reaction was performed in 15 μl volume, containing 1.5 μl Taqman RT Buffer (10 × ), 0.15 μl 100 mM dNTPs (100 mM), 1.0 μl Reverse Transcriptase, 0.19 μl RNase inhibitor (20 U μl^−1^), 3.0 μl specific miRNA primer, 100 ng total RNA and nuclease-free water. Real-time qPCR was performed in 20 μl reaction volume using the standard protocols on an Applied Biosystems 7900HT System. Briefly, 2.5 μl of cDNA was mixed with 10 μl TaqMan universal PCR master mix (2 × ), 1.0 μl TaqMan miRNA assay and 6.5 μl nuclease-free water. The reaction conditions were the same as above for real-time PCR in array experiments. For each RNA sample, the target miRNA and RNU48 reactions were run in triplicate.

Mimics of miR-32 and -590-5p or control (50 nM) were transfected into cells using the Lipofectamine RNAiMAX (Invitrogen) according to the manufacturer's protocol. Anti-miRs against miR-32 and -590-5p mercury LNA or control (50 nM) were similarly introduced into cells. The sequences of mimics and the anti-miRs are given below.

miRIDIAN microRNA Mimics

hsa-miR-32-5p: 5′-UAUUGCACAUUACUAAGUUGCA-3′

hsa-miR-590-5p: 5′-GAGCUUAUUCAUAAAAGUGCAG-3′

Negative control: 5′-UUGUACUACACAAAAGUACUG-3′

miRCURY LNA inhibitor

hsa-miR-32-5p: 5′-CAACTTAGTAATGTGCAAT-3′

hsa-miR-590-5p: 5′-CTGCACTTTTATGAATAAGCT-3′

Negative control: 5′-AGAGCTCCCTTCAATCCAAA-3′

### 3′-UTR plasmid reporter assay

pLenti-UTR-GFP plasmid, in which the 3′-UTR of BTG2 is inserted as the 3′-UTR of GFP, was purchased from Applied Biological Materials Inc. The 3′-UTR of BTG2 lacking the binding sites for miR-32 and -590-5p was derived from this plasmid using the QuickChange Lightning Site-Directed Mutagenesis Kit (Agilent Technologies) according to the manufacturer's protocol. 293T human embryonic kidney cells were transfected with both the wild-type and the miR-binding site-deleted pLenti-UTR plasmids using X-tremeGENE HP DNA transfection reagent (Roche Applied Science). Cells were transfected with miRNAs (miR-32, miR-590-5p or control) using Lipofectamine RNAiMAX, 48 h after transfection, with pLenti-UTR-GFP and were analysed with MACS Flow Cytometry (Miltenyl Biotec) for the mean fluorescent intensity 72 h after transfection of the miR.

### RNA interference assay

siBTG2 sequence (5′-CAGAGCACUACAAACACCACUGGUU-3′), siWDR82#1 sequence (5′-UCUCAUCUGUAGUUUCCAATT-3′), siWDR82#2 sequence (5′-GAUCCAGAAGGGUUAAUUUTT-3′) or control (50 nM) were transfected into cells using the Lipofectamine RNAiMAX (Invitrogen) according to the manufacturer's protocol.

### Cell cycle analysis

Cells were harvested, washed twice in PBS, resuspended in 0.1% saponin/PBS (Sigma) containing 10 μg ml^−1^ RNase (Sigma) and 50 μg ml^−1^ propidium iodide (Sigma) and incubated for 30 min at 37 °C. Samples were analysed with flow cytometry using the FACScalibur and CellQuest software (Becton Dickinson).

### *In vivo* tumorigenesis assay

DU145 cells (1 × 10^6^) infected with shGFP and shSETD1A lentiviruses (in 100 μl of 1:1 PBS and matrigel (BD Biosciences)) were separately inoculated subcutaneously into the flanks on either side of 6- to 8-week-old male athymic nude mice (*n*=3 for control; *n*=6 for experimental group). Tumours were measured every week and tumour volume was calculated as length × width^2^ × 0.52.

For MDA-MB-231 xenografts, 6-week-old female athymic nude mice (*n*=6 for control group, *n*=10 for experimental group) were anaesthetized with isofluorane, and luciferase-tagged tumour cells (1 × 10^6^) infected with shGFP and shSETD1A lentiviruses (1 × 10^6^) in 100 μl of 1:1 PBS and Matrigel (BD) were injected into the fourth right mammary fat pad. Tumour-derived signals were monitored by *in vivo* live imaging using IVIS Lumina II (PerkinElmer). D-Luciferon substrate (PerkinElmer) was injected intraperitoneally at 150 μl per animal; mice were anaesthetized after 5 min; and bioluminescent images were taken.

All mice were cared for and experiments were performed under the AAALAS guidelines using protocols approved by the institutional review board and the institutional animal care and use committee of the Massachusetts General Hospital.

### Arrayed gene expression analysis

RNA expression in SETD1A-depleted A549 and MDA-MB-231 cells was analysed using the Human Gene Expression 12 × 135 K Arrays (GPL17425, Roche Nimblegen). Briefly, RNA was extracted from A549 and MDA-MB-231 cells infected with shGFP or two independent shSETD1A constructs (shSETD1A#1 and shSETD1A#2) using the RNeasy Mini kit (Qiagen). cDNA synthesis was performed using the Roche cDNA synthesis system (11 117 831 001). cDNA was hybridized to the Human Gene Expression 12 × 135 K Arrays in duplicate according to the manufacturer's protocol. The microarrays were scanned on Nimblegen MS200 at 2 μm resolution. Scans were converted to robust microarray average (RMA)-normalized expression values using the Roche NimbleScan 2.6 software.

The list of upregulated genes common to MDA-MB-231 cells infected with shSETD1A#1 and shSETD1A#2 was compiled. The 897 genes induced in SETD1A-depleted cells thus identified were analysed against the 80 miRNAs commonly suppressed in MDA-MB-231 cells infected with shSETD1A#1 and shSETD1A#2. The 185 miRNA–mRNA interactions predicted by the three miRNA target predictor programmes, TargetScan, miRanda and PicTar, were then compared with the list of common genes upregulated in A549 cells infected with shSETD1A#1 and SETD1A#2. This analysis identified 31 genes to be common targets of SETD1A-regulated miRNAs. These 185 and 31 genes were then subjected to pathway enrichment analysis (GSEA). The gene set enrichment hypergeometric tests between the 185 miRNA targets and the curated gene lists (C2) of MSigDB were accomplished using the investigate gene sets tool (http://www.broadinstitute.org/gsea/msigdb/annotate.jsp; top 100 gene sets; FDR=0.05). When noted, GSEA was performed by the Broad Institute (Cambridge, MA, USA). *P* values were generated using hypergeometric GSEA applied to the each subset of Broad's MSigDB gene set database. The Benjamini–Hochberg method was used to compute FDR *q*-values from the *P* values.

### Oncomine microarray data sets

Oncomine data sets were analysed for differences in SETD1A mRNA between cancer and normal tissues (gene rank top 20%; *P* value<0.005, Student's *t*-test). Representative box plot from Oncomine (www.oncomine.org) is shown to illustrate the difference in SETD1A mRNA levels. All data are log-transformed and median-centred, and the 25th–75th percentiles are indicated by the closed box.

### Statistical analysis

Statistical analysis was performed with a two-tailed Student's *t*-test or as described in each method by the Broad Institute. The differences between the means were considered to be statistically significant at *P*<0.05.

## Additional information

**How to cite this article:** Tajima, K. *et al.* SETD1A modulates cell cycle progression through a miRNA network that regulates p53 target genes. *Nat. Commun.* 6:8257 doi: 10.1038/ncomms9257 (2015).

## Supplementary Material

Supplementary Information Supplementary Figures 1-8, Supplementary Tables 1-6 and Supplementary References
