# Supplementary material for: SETD1A modulates cell cycle progression through a miRNA network that regulates p53 target genes
Source: Nat Commun. 2015 Sep 23;6:8257. doi: 10.1038/ncomms9257 (PMC4667427; doi:10.1038/ncomms9257)
Supplement: Supplementary Information — Supplementary Figures 1-8, Supplementary Tables 1-6 and Supplementary References [file ncomms9257-s1.pdf]

Supplementary Information

Supplementary Figures

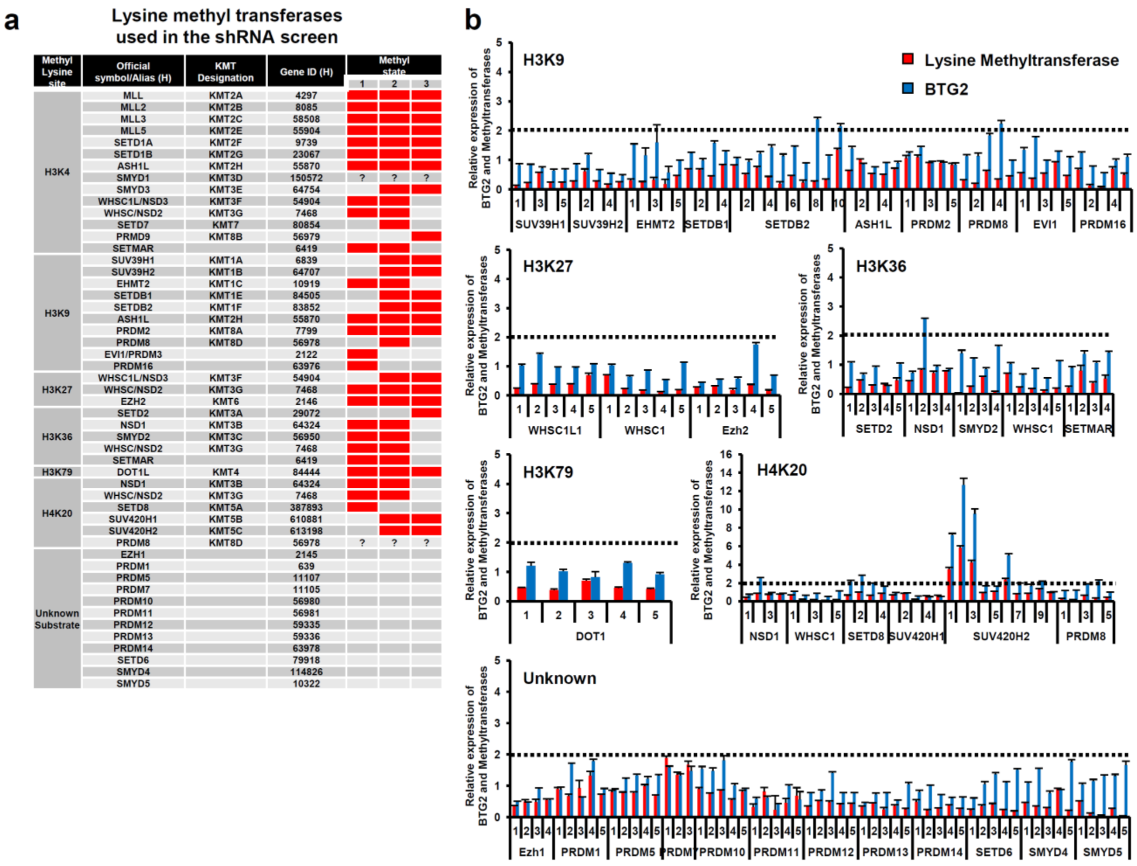

Supplementary Figure 1.

a) List of KMTs targeted in the shRNA screen. The official symbol, KMT designation, gene ID and specifities are provided. Those highlighted in red have the potential to methylate the indicated methylation state. (?) denotes the ability to modify indicated residue whereas the methylation state is unknown.

b) A shRNA screen against KMTs to identify the chromatin modifiers that regulate BTG2 expression. Lenti-virally expressed shRNA screen against the KMTs shown was performed using MDA-MB-231 cells. pLKO infected cells were used as control and the expression of BTG2 and SETD1A in these cells was set at 1. The fold change in BTG2

and each KMT expression after 72 hr of viral infection is shown. The dotted line marks 2 fold induction of BTG2 compared to control. KMTs and BTG2 are shown as red and blue bars, respectively. Data are represented as mean ± s.d. of the average of 3 experimental replicates.

a

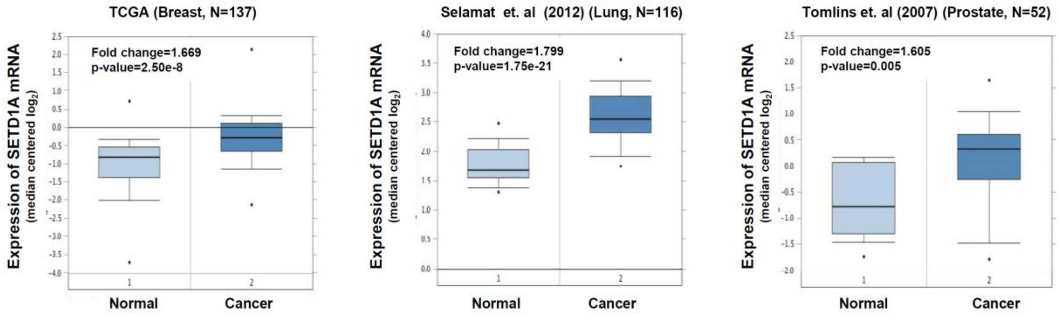

b

Tissue type	Cell line	SETD1A Fold suppression	BTG2 Fold induction
Breast	MCF7 *	0.19±0.05	5.37±0.12
	MDA-MB-468	0.37±0.02	4.7±0.18
	BT549	0.32±0.02	1.51±0.04
Lung	H1299	0.20±0.01	1.52±0.17
	A549 *	0.35±0.02	10.69±0.18
Colon	HCT8 *	0.34±0.01	3.75±0.14
	H630	0.24±0.01	1.98±0.11
	HCT116 *	0.36±0.01	2.38±0.06
	DLD1	0.45±0.01	1.40±0.06
	SW620	0.43±0.03	2.24±0.07
	HT29	0.18±0.01	4.60±0.19
Prostate	DU145	0.39±0.01	2.31±0.07
	LNCaP*	0.15±0.01	3.21±0.11

* Wt p53

c

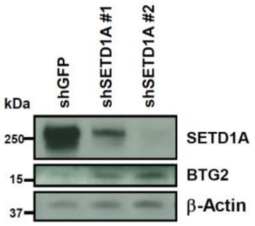

Supplementary Figure 2.

a) SETD1A mRNA expression is elevated in breast, lung and prostate cancers compared to normal tissues. Box plots from the studies indicated are shown[1, 2]. TCGA : The Cancer Genome Atlas[3]. *P*-value is determined by Student's *t*-test and representative box plot is shown to illustrate the difference in SETD1A mRNA levels. All data are log transformed, median centered and the 25th - 75th percentiles are indicated by the closed box.

b) A list of cell lines in which SETD1A was knocked down with shSETD1A#1 and shSETD1A#2 lentiviruses. The expression of SETD1A and BTG2 was analyzed by qPCR. The fold suppression of SETD1A and induction of BTG2 in each cell line is shown. SETD1A and BTG2 expression in SETD1A depleted cells represent the average derived from cells individually infected with two shSETD1A constructs (shSETD1A#1 and shSETD1A#2). shGFP infected cells were used as control and the average

expression of BTG2 and SETD1A in these cells was set at 1. Cells lines harboring wild type p53 are marked with an asterisk. Data are represented as mean ± s.d. of the average of 3 experimental replicates.

c) Western blot analysis of MDA-MB-231 cells infected with shGFP and two shSETD1A constructs demonstrates the depletion of SETD1A and the concomitant increase in BTG2. ß-actin was used as loading control.

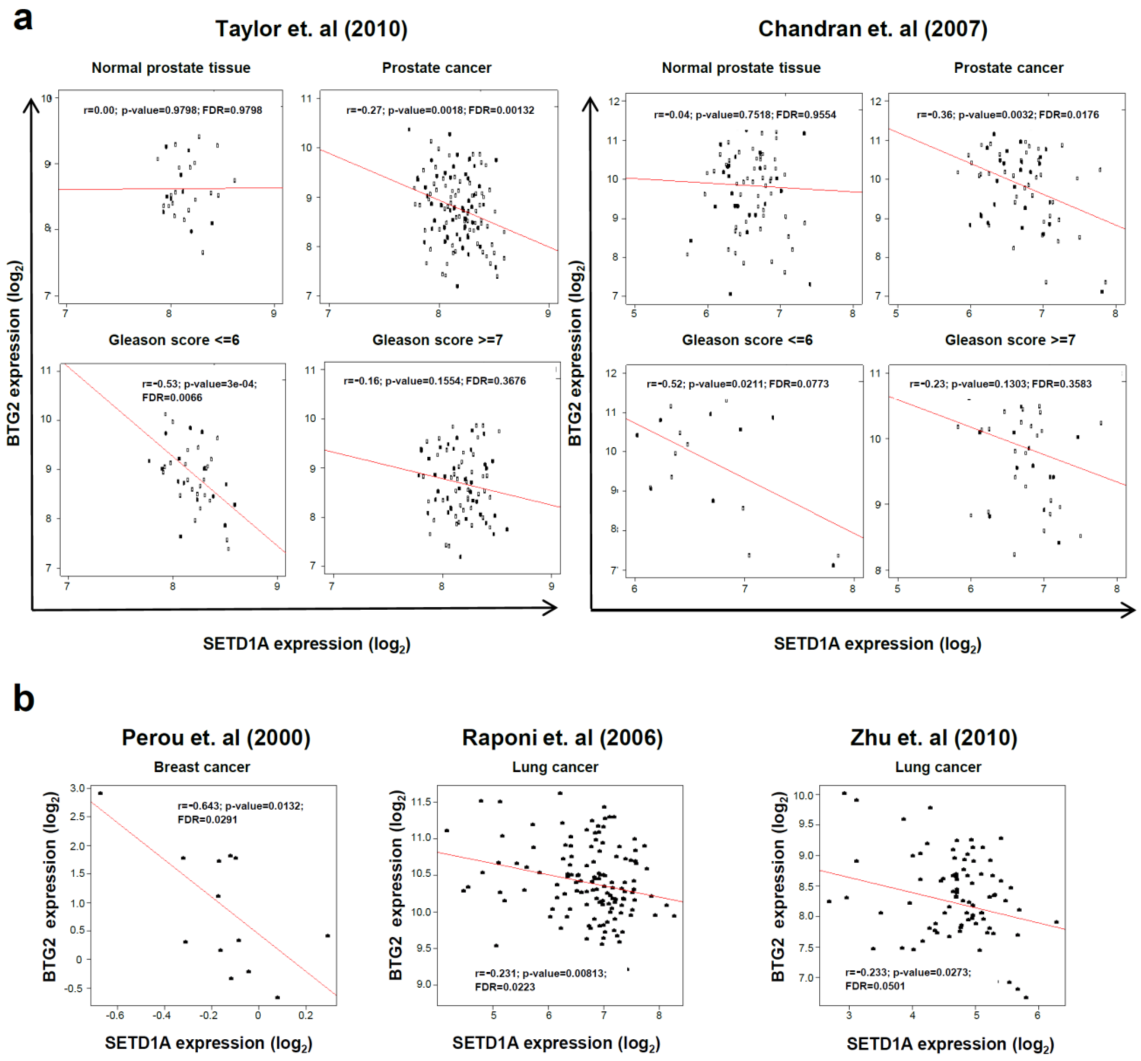

Supplementary Figure 3.

a) Pearson correlation of SETD1A and BTG2 expression in a large prostate cancer and normal tissue data set[4, 5] demonstrates significant inverse correlation between SETD1A and BTG2 expression in tumors, which is pronounced in tumors of Gleason Score ≤ 6. No correlation was detected in the normal tissue samples.

b) Pearson correlation of SETD1A and BTG2 expression in breast and lung cancer data sets[6, 7, 8] demonstrate significant inverse correlation between SETD1A and BTG2 expression.

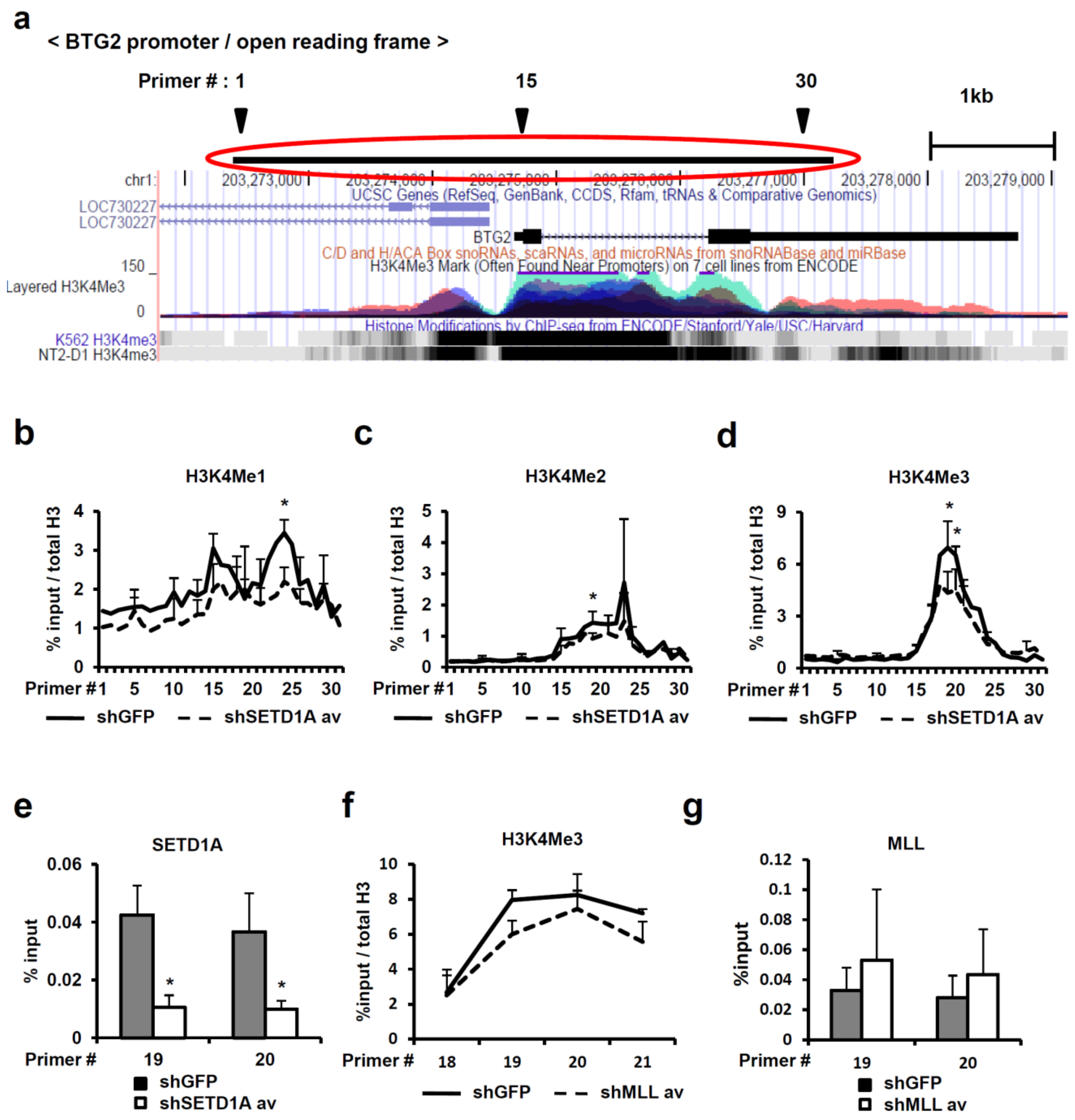

Supplementary Figure 4.

a, b, c & d) Thirty one, 200 bp oligonucleotides spanning 3 kb upstream and 2 kb down stream of the BTG2 open reading frame (a) were analyzed by ChIP for H3K4Me1 (b), H3K4Me2 (c), and H3K4Me3 (d) marks. The amplitude of the various marks across the region was standardized against input and total H3 levels in control and SETD1A knockdown MDA-MB-231 cells. The regions demonstrating significant differences in H3K4 methylation are marked with asterisks.

e) SETD1A binding to the BTG2 promoter region demonstrating significant changes in H3K4Me3 peak analyzed with primers 19 and 20 is shown.

f) MLL depletion does not change the H4K3Me3 peaks in the BTG2 promoter region analyzed with primers 18 through 21.

g) MLL binding to the BTG2 promoter region analyzed with primers 19 and 20 shows no significant changes in MLL binding to these sites.

For all experiments, the shSETD1A and shMLL data represent the average derived from ChIP assays performed with MDA-MB-231 cells individually infected with two shSETD1A constructs (shSETD1A#1 and shSETD1A#2) or two shMLL constructs. Data are represented as mean ± s.d. of the average of 3 experimental replicates. Asterisks indicate *P* values of (P<0.05) for relevant figures by Student's *t*-test.

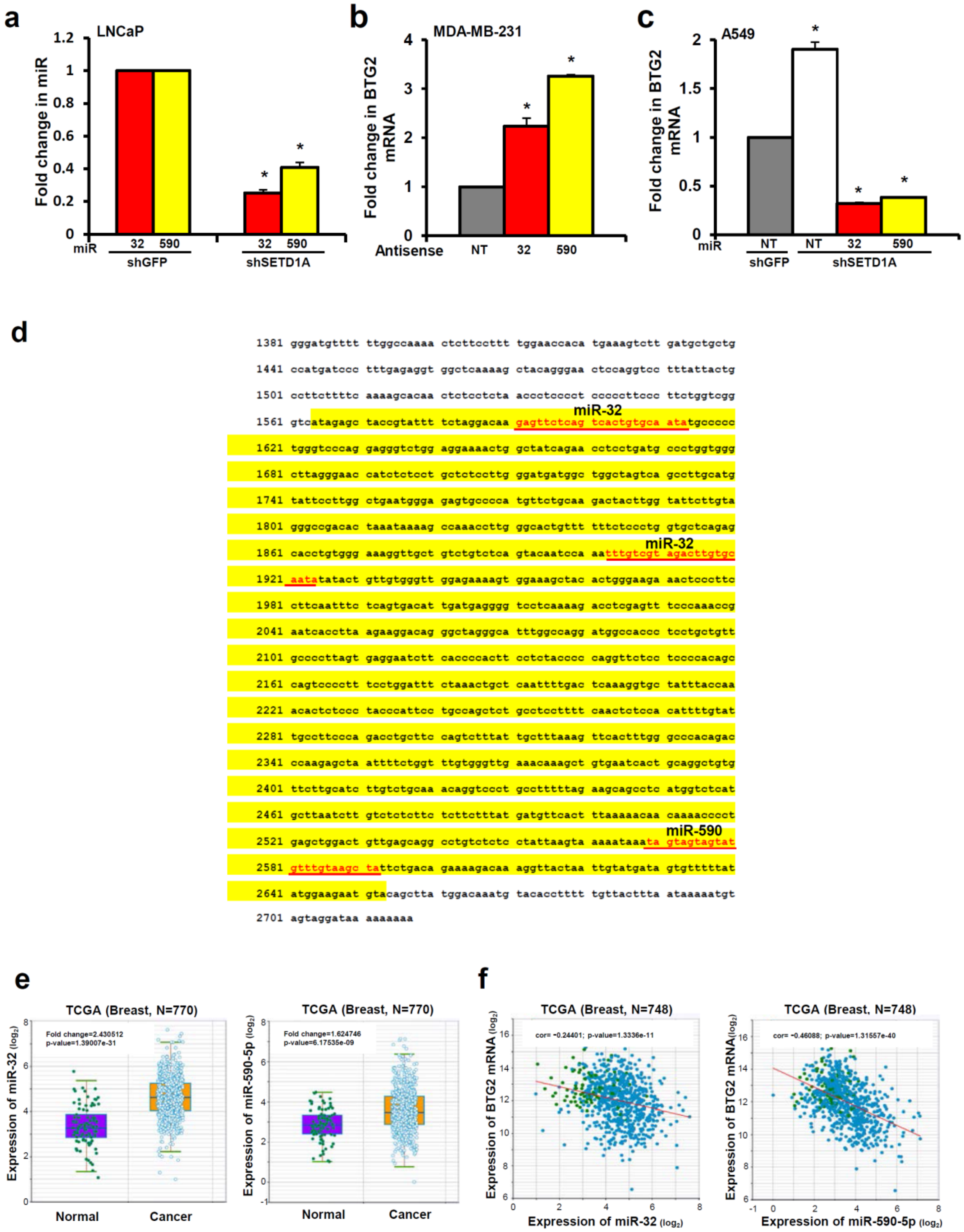

Supplementary Figure 5.

a) Expression of miRNA-32 and -590-5p was analyzed in the prostate cancer cell line LNCaP by qPCR. The expression of miRNAs in shGFP infected control cells was set at 1.

The miRNA expression in SETD1A depleted cells represents an average derived from LNCaP cells individually infected with two different shSETD1A constructs (shSETD1A#1 and shSETD1A#2).

b) Suppression of miRNA-32 and -590-5p, induces BTG2 expression. Level of BTG2 in MDA-MB-231 cells transfected with non-targeting sequences (NT) was set at 1.

c) Expression of miRNA-32 and -590-5p abrogates BTG2 induction in SETD1A depleted A549 cells. SETD1A expression in shGFP infected control cells was set at 1.

For figures (a), (b) and (c): NT represents Non-targeting and 590 indicates miR-590-5p. Data are represented as mean ± s.d. of the average of 3 experimental replicates. Asterisks indicate *P* values of ($P<0.05$) for relevant figures by Student's *t*-test.

d) The sequence of the 3'UTR of BTG2 mRNA. The positions of the two miRNA-32 binding sites and the one miRNA-590 binding site are shown. The deletion construct which lacks miRNA-32 and -590 binding sites was generated by removing the sequences highlighted in yellow.

e) Analysis of a large breast cancer data set (The Cancer Genome Atlas (TCGA) [3]; n=770) shows increased expression of miRNA-32 and -590-5p in tumors compared with normal tissue. *P*-value is determined by Student's *t*-test and representative box plot is shown to illustrate the difference in the level of each miRNA. All data are log transformed, median centered and the 25th - 75th percentiles are indicated by the closed box.

f) Pearson correlation of miR-32 and -590-5p and BTG2 expression in TCGA[3] (n=748) demonstrates significant inverse correlation between the miRNAs and BTG2. Analysis was performed by Starbase v2.0[9, 10].

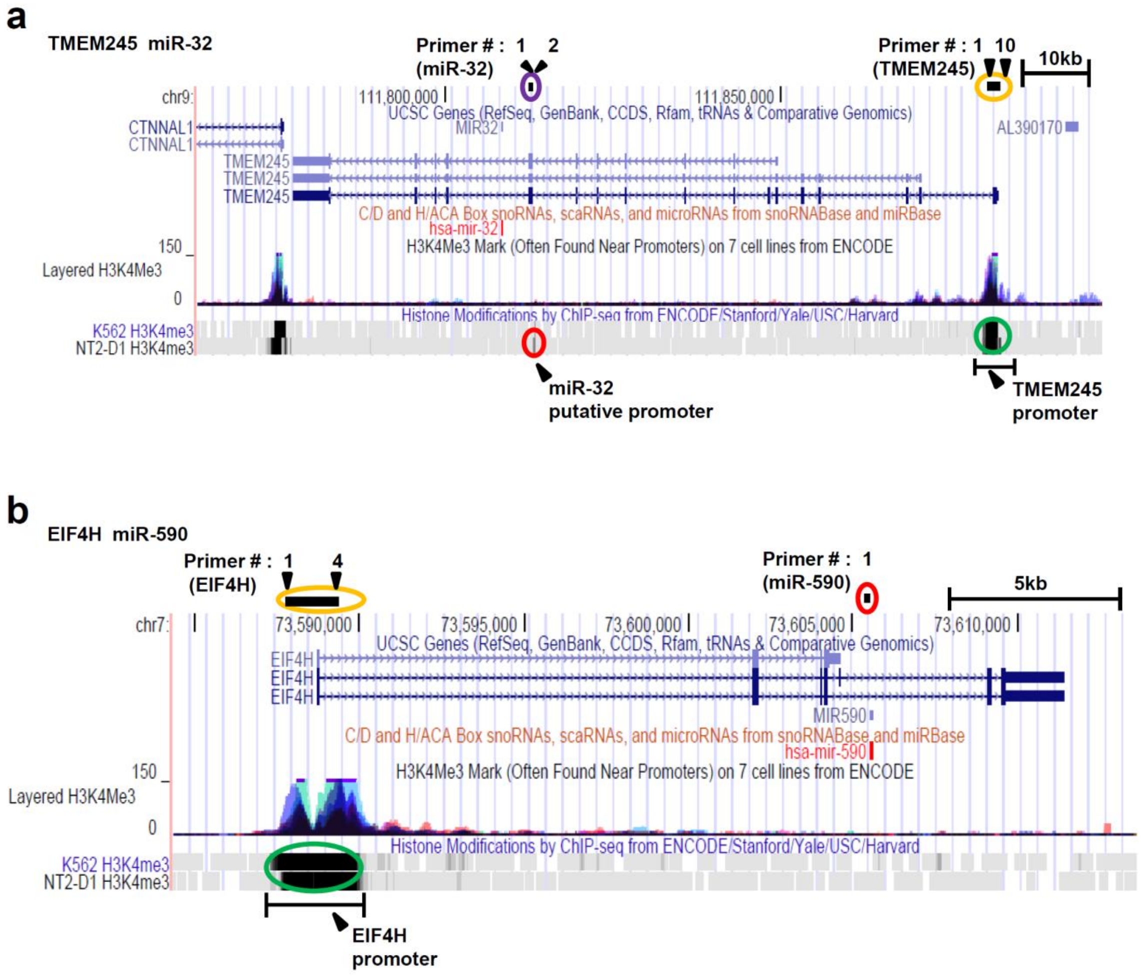

Supplementary Figure 6.

a) A screen shot of the UCSC genome browser shows the genomic region harboring miR-32, which is embedded within the host gene TMEM245 (http://genome.ucsc.edu). The promoter of TMEM245 and the putative promoter of miR-32 are marked with green and red ovals, respectively. Note the H3K4 methylation patterns in these two regions. ChIP assays were performed using 10 and 2 primers spanning the TMEM245 (yellow oval) and miR-32 (purple oval) promoter regions (indicated with arrows), respectively (Fig. 3a, 3b).

b) A screen shot of the UCSC genome browser shows the genomic region harboring miR-590, which is embedded within the host gene EIF4H. The EIF4H promoter is marked

with a green oval. The red oval represents a region within the previously defined miR-590 promoter[11], which was further analyzed by ChIP assays. ChIP assays were performed using 4 and 1 primer(s) spanning the EIF4H (yellow oval) and miR-590 promoter regions (indicated with arrows), respectively (Fig. 3c, 3d).

a

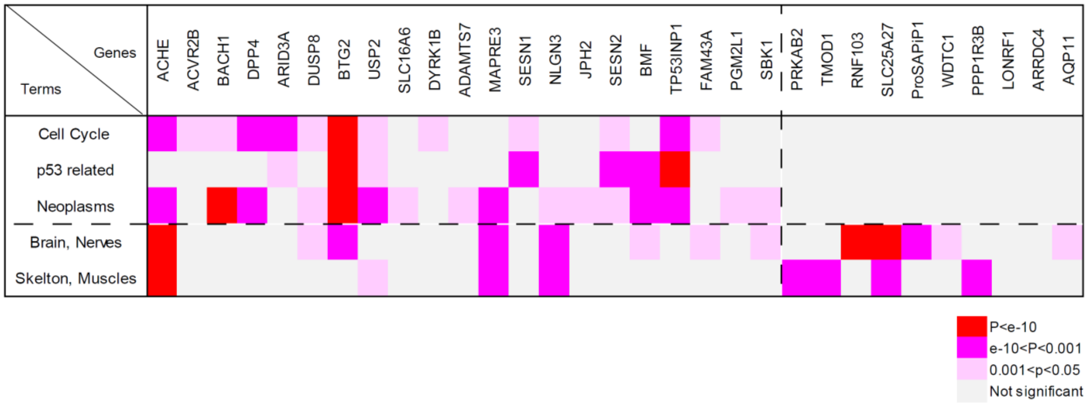

b

Term Id	Term Name	P-Value	FDR
GO:0008285	negative regulation of cell proliferation	5.19E-13	1.05E-10
GO:0007049	cell cycle	2.42E-10	3.55E-08
GO:0007050	cell cycle arrest	1.45E-08	1.51E-06
GO:0000080	G1 phase of mitotic cell cycle	9.68E-07	7.43E-05
hsa05200	pathways in cancer	5.24E-15	1.252E-12
hsa04110	cell cycle	9.90E-07	1.076E-05
hsa04115	p53 signaling pathway	4.72E-06	3.522E-05
DOID:162	in tumors	7.36E-16	4.84E-13
DOID:4241	in breast cancer	1.93E-14	6.36E-12
DOID:10283	in prostate cancer	5.977E-09	7.865E-07
DOID:2619	metastasis	3.274E-06	0.0002393

Supplementary Figure 7.

a) The heat map derived using the Gene, Disease Features Ontology-based Overview System[12, 13] (http://gendoo.dbcls.jp/) demonstrates the profile of the 31 targets genes common to both MDA-MB-231 and A549 cells modulated by SETD1A-regulated miRNAs. Twenty one of the 31 genes are significantly related to cell cycle, p53 pathway, and neoplasms while the others are also related to the nervous and musculoskeletal system. The colors highlight the degree of significance associated with each gene in relation to the pathways shown and denote *P*-values described in the figure by Mesh terms enrichment analysis using Biocompass software, under the Creative Commons Attribution 2.1 Japan License.

b) StarBase enrichment analysis of the functional terms for miRNA targets shows that the 24 SETD1A induced miRNAs which target the 21 genes (see main text) are significantly

enriched for the pathways shown. *P*-values are obtained from a hypergeometric test and Bonferroni/FDR correction.

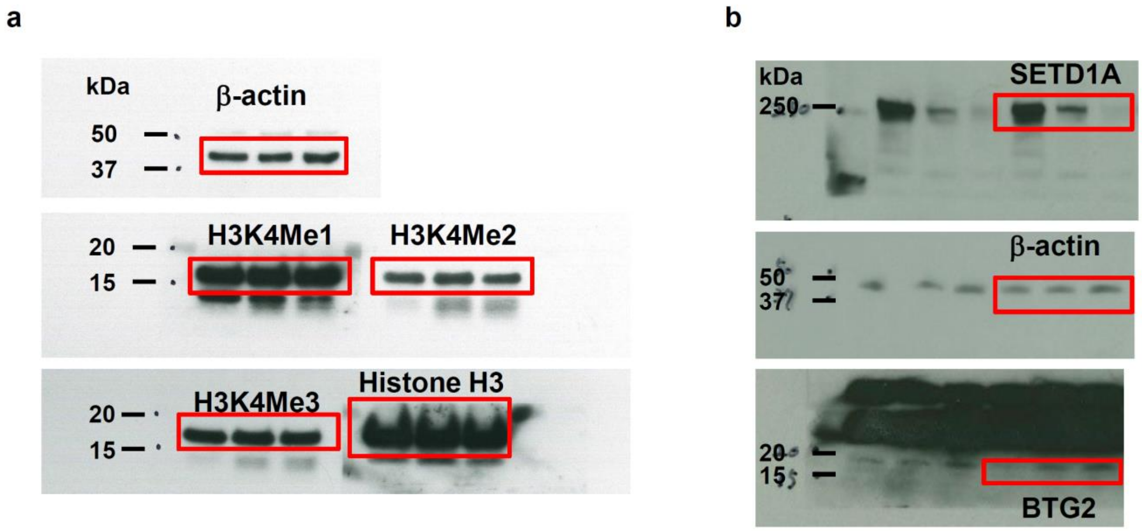

Supplementary Figure 8.

a)Uncropped blots from Figure 1b.

b)Uncropped blots from Supplementary Figure 2c

Supplementary Tables

miRNAs down-regulated (suppressed by 60%) in SETD1A depleted MDA-MB-231	
miRNA	
hsa-miR-338-5P-002658	hsa-miR-362-3p-4395228
hsa-miR-1274B-002884	hsa-miR-106b-4373155
hsa-miR-1274A-002883	hsa-miR-345-4395297
hsa-miR-1305-002867	hsa-miR-942-002187
hsa-miR-27a#-002445	hsa-miR-204-4373094
hsa-miR-30e-3p-000422	hsa-miR-301b-4395503
hsa-miR-625#-002432	hsa-miR-886-5p-4395304
hsa-miR-30a-5p-000417	hsa-miR-598-4395179
hsa-miR-1180-002847	hsa-miR-106a-4395280
hsa-miR-25#-002442	hsa-miR-23b-4373073
hsa-miR-424#-002309	hsa-miR-660-4380925
hsa-miR-20a#-002437	hsa-miR-26b-4395167
hsa-miR-106b#-002380	hsa-miR-15b-4373122
hsa-miR-664-002897	hsa-miR-186-4395396
hsa-miR-125b-1#-002378	hsa-miR-628-5p-4395544
hsa-miR-505#-002087	hsa-miR-20b-4373263
hsa-miR-296-5p-4373066	hsa-miR-454-4395434
hsa-miR-589-4395520	hsa-miR-99a-4373008
hsa-miR-147b-4395373	hsa-miR-29b-1#-002165
hsa-miR-148b-4373129	hsa-miR-222-4395387
hsa-miR-10b-4395329	hsa-miR-885-5p-4395407
hsa-miR-330-3p-4373047	hsa-miR-1270-002807
hsa-miR-222#-002097	hsa-miR-199a-3p-4395415
hsa-miR-636-4395199	hsa-miR-142-3p-4373136
hsa-miR-545-4395378	hsa-miR-618-4380996
hsa-miR-328-4373049	hsa-miR-17-4395419
hsa-miR-590-5p-4395176	hsa-miR-301a-4373064
hsa-miR-190-4373110	hsa-miR-24-4373072
hsa-miR-1255B-002801	hsa-miR-324-3p-4395272
hsa-miR-744-4395435	hsa-miR-126-4395339
hsa-miR-34a-4395168	hsa-miR-532-3p-4395466
hsa-miR-139-5p-4395400	hsa-miR-223-4395406
hsa-miR-139-3p-4395424	hsa-miR-93-4373302
hsa-miR-9-4373285	hsa-let-7e-4395517
hsa-miR-107-4373154	hsa-miR-135b-4395372
hsa-miR-452-4395440	hsa-miR-505-4395200
hsa-miR-152-4395170	hsa-miR-32-4395220
hsa-miR-219-1-3p-4395206	hsa-miR-18a-4395533
hsa-miR-423-5p-4395451	hsa-miR-29b-4373288
hsa-miR-34a#-002316	hsa-miR-320a-4395388

miRNAs up-regulated (>2 fold) in SETD1A depleted MDA-MB-231	
miRNA	
hsa-miR-1236-002761	hsa-miR-497-001043
hsa-miR-938-002181	hsa-miR-545#-002266
hsa-let-7e#-002407	hsa-miR-432-001026
hsa-let-7g#-002118	hsa-miR-125b-2#-002158
hsa-miR-214#-002293	hsa-miR-193b#-002366
hsa-let-7c#-002405	hsa-miR-18a#-002423
hsa-miR-302d-000535	hsa-miR-1254-002818
hsa-miR-200a#-001011	hsa-let-7i#-002172
hsa-miR-643-001594	hsa-miR-130b#-002114
hsa-miR-1285-002822	hsa-miR-29b-2#-002166
hsa-let-7f-2#-002418	hsa-miR-517a-4395513
hsa-miR-33a-002135	hsa-miR-744#-002325
hsa-miR-1267-002885	hsa-miR-1243-002854
hsa-let-7a#-002307	hsa-miR-519a-4395526
hsa-miR-939-002182	hsa-miR-202-4395474
hsa-miR-181a-2#-002317	hsa-miR-615-5p-4395464
hsa-miR-99a#-002141	hsa-miR-30c-2#-002110
hsa-miR-191#-002678	hsa-miR-151-5P-002642
hsa-miR-10a#-002288	hsa-miR-206-000510
hsa-miR-616-001589	hsa-miR-34c-5p-4373036
hsa-miR-1257-002910	hsa-miR-941-002183
hsa-miR-192#-002272	hsa-miR-302a-4378070
hsa-miR-7-2#-002314	hsa-miR-221#-002096
hsa-miR-213-000516	hsa-miR-576-5p-4395461
hsa-let-7f-1#-002417	hsa-miR-522-4395524
hsa-let-7b#-002404	hsa-miR-1201-002781
hsa-miR-1227-002769	hsa-miR-184-4373113
hsa-miR-17#-002421	hsa-miR-517c-4373264
hsa-miR-1303-002792	hsa-miR-425#-002302
hsa-miR-1291-002838	hsa-miR-1271-002779
hsa-miR-340#-002259	hsa-miR-100#-002142
dme-miR-7-000268	hsa-miR-622-001553
hsa-miR-1269-002789	hsa-miR-30d-000420
hsa-miR-1244-002791	

Supplementary Table 1. List of miRNAs induced by >2 fold or suppressed by 60% in SETD1A-depleted MDA-MB-231 cells compared to shGFP expressing MDA-MB-231 cells.

miRNA target genes up-regulated in SETD1A depleted MDA-MB-231

GeneID	GeneName
NM_015831	ACHE
NM_001106	ACVR2B
NM_003183	ADAM17
NM_014272	ADAMTS7
NM_000684	ADRB1
NM_173039	AQP11
NM_005224	ARID3A
NM_006465	ARID3B
NM_183376	ARRDC4
NM_004655	AXIN2
NM_001186	BACH1
NM_012342	BAMBI
NM_001003940	BMF
NM_153252	BRWD3
NM_006763	BTG2
NM_001010911	C10orf114
NM_022153	C10orf54
NM_001001436	C16orf87
NM_175063	C19orf63
NM_020130	C8orf4
NM_152765	C8orf46
NM_001013842	C8orf58
NM_014333	CADM1
NM_001221	CAMK2D
NM_001759	CCND2
NM_003885	CDK5R1
NM_004064	CDKN1B
NM_020770	CGN
NM_024111	CHAC1
NM_015557	CHD5
NM_017780	CHD7
NM_001286	CLCN6
NM_021101	CLDN1
NM_148960	CLDN19
NM_001831	CLU
NM_000090	COL3A1
NM_033641	COL4A6
NM_000093	COL5A1
NM_182485	CPEB2
NM_014912	CPEB3
NM_181571	CREM
NM_004382	CRHR1
NM_033027	CSRNP1
NM_001338	CXADR
NM_020311	CXCR7
NM_004734	DCLK1
NM_144973	DENND5B
NM_021240	DMRT3
NM_015569	DNM3
NM_001935	DPP4
NM_006426	DPYSL4
NM_004420	DUSP8
NM_004714	DYRK1B
NM_001951	E2F5
NM_022073	EGLN3
NM_001964	EGR1
NM_001420	ELAVL3
NM_198573	ENHO
NM_001247	ENTPD6
NM_005442	EOMES
NM_020526	EPHA8
NM_004443	EPHB3

GeneID	GeneName
NM_001432	EREG
NM_024896	ERMP1
NM_001991	EZH1
NM_004101	F2RL2
NM_153690	FAM43A
NM_017633	FAM46A
NM_003862	FGF18
NM_004472	FOXD1
NM_014728	FRMPD4
NM_012193	FZD4
NM_198460	GBP6
NM_138426	GLCCI1
NM_014905	GLS
NM_002078	GOLGA4
NM_152742	GPC2
NM_019593	GPCPD1
NM_003272	GPR137B
NM_001006636	GTDC1
NM_015094	HIC2
NM_003959	HIP1R
NM_031935	HMCN1
NM_002130	HMGCS1
NM_005522	HOXA1
NM_002166	ID2
NM_006547	IGF2BP3
NM_000599	IGFBP5
NM_002181	IHH
NM_000575	IL1A
NM_032594	INSM2
NM_001005909	IP6K2
NM_000214	JAG1
NM_020433	JPH2
NM_002235	KCNA6
NM_015299	KHNYN
NM_003709	KLF7
NM_030915	LBH
NM_012317	LDOC1
NM_018113	LMBR1L
NM_152271	LONRF1
NM_004525	LRP2
NM_052886	MAL2
NM_003954	MAP3K14
NM_005204	MAP3K8
NM_002748	MAPK6
NM_012326	MAPRE3
NM_002403	MFAP2
NM_004225	MFHAS1
NM_002405	MFNG
NM_152291	MUC7
NM_006403	NEDD9
NM_033119	NKD1
NM_018977	NLGN3
NM_000901	NR3C2
NM_004822	NTN1
NM_021229	NTN4
NM_014840	NUAK1
NM_001031716	OBFC2A
NM_145260	OSR1
NM_173582	PGM2L1
NM_017933	PID1
NM_014798	PLEKHM1
NM_004073	PLK3

GeneID	GeneName
NM_002699	POU3F1
NM_000943	PPIC
NM_024607	PPP1R3B
NM_005399	PRKAB2
NM_014731	ProSAPiP1
NM_175887	PRR15
NM_007039	PTPN21
NM_171998	RAB39B
NM_014636	RALGPS1
NM_020211	RGMA
NM_004296	RGS6
NM_004040	RHOB
NM_005667	RNF103
NM_012424	RPS6KC1
NM_021244	RRAGD
NM_001024401	SBK1
NM_004866	SCAMP1
NM_014575	SCHIP1
NM_006922	SCN3A
NM_015187	SEL1L3
NM_006216	SERPINE2
NM_014454	SESN1
NM_031459	SESN2
NM_170600	SH2D3C
NM_017699	SIDT1
NM_173354	SIK1
NM_020808	SIPA1L2
NM_004694	SLC16A6
NM_004277	SLC25A27
NM_080552	SLC32A1
NM_019849	SLC7A10
NM_005904	SMAD7
NM_032840	SPRYD3
NM_006011	ST8SIA2
NM_003714	STC2
NM_177424	STX12
NM_032701	SUV420H2
NM_014849	SV2A
NM_003179	SYP
NM_205848	SYT6
NM_015130	TBC1D9
NM_080751	TMC2
NM_001017395	TMCC1
NM_018426	TMEM63B
NM_001008495	TMEM64
NM_003275	TMOD1
NM_015028	TNIK
NM_033285	TP53INP1
NM_000368	TSC1
NM_014779	TSC22D2
NM_003565	ULK1
NM_004205	USP2
NM_020335	VANGL2
NM_003383	VLDLR
NM_080607	VSTM2L
NM_015023	WDTC1
NM_145166	ZBTB47
NM_033089	ZCCHC3
NM_020951	ZNF529
NM_021216	ZNF71
NM_178835	ZNF827

Supplementary Table 2. miRNA/mRNA interactions of genes and miRNAs differentially expressed between shGFP-MDA-MB-231 control and sh-SETD1A-MDA-MB-231 cells identified using TargetScan, miRanda and PicTar. The high confidence set of consensus miRNA targets identified by all three algorithms were selected. The 185 genes induced by SETD1A-regulated miRNAs are shown.

Gene Set Name	Description	Source	Genes	Overlap	p-value	FDR
PEREZ TP53 TARGETS	Genes up-regulated in the HMEC cells (primary mammary epithelium) upon expression of TP53 [GeneID=7157] off adenoviral vector.	MSigDB	1174	39	1.63E-24	1.04E-20
PEREZ TP63 TARGETS	Genes up-regulated in the HMEC cells (primary mammary epithelium) upon expression of the transcriptionally active isoform of TP63 [GeneID=8626] off adenoviral vector.	MSigDB	355	24	2.32E-22	7.39E-19
GOZGIT ESR1 TARGETS DN	Genes down-regulated in TMX2-28 cells (breast cancer) which do not express ESR1 [GeneID=2099]) compared to the parental MCF7 cells which do.	MSigDB	781	29	1.26E-19	2.68E-16
NUYTTEN EZH2 TARGETS UP	Genes up-regulated in PC3 cells (prostate cancer) after knockdown of EZH2 [GeneID=2146] by RNAi.	MSigDB	1037	32	3.15E-19	4.43E-16
BUYTAERT PHOTODYNAMIC THERAPY STRESS UP	Genes up-regulated in T24 (bladder cancer) cells in response to the photodynamic therapy (PDT) stress.	MSigDB	811	29	3.48E-19	4.43E-16
PEREZ TP53 AND TP63 TARGETS	Genes up-regulated in HMEC cells (primary mammary epithelium) upon expression of both of TP53 [GeneID=7157] and the transcriptionally active isoform of TP63 [GeneID=8626] off adenoviral vectors.	MSigDB	205	18	4.63E-19	4.91E-16
DUTERTRE ESTRADIOL RESPONSE 24HR DN	Genes down-regulated in MCF7 cells (breast cancer) at 24 h of estradiol [PubChemID=5757] treatment.	MSigDB	505	23	1.18E-17	1.07E-14
ZWANG CLASS 1 TRANSIENTLY INDUCED BY EGF	Class I of genes transiently induced by EGF [GeneID =1950] in 184A1 cells (mammary epithelium).	MSigDB	516	21	3.06E-15	2.43E-12
STK33 UP	Genes up-regulated in NOMO-1 and SKM-1 cells (AML) after knockdown of STK33 [Gene ID=65975] by RNAi.	MSigDB	293	17	4.78E-15	3.38E-12
KINSEY TARGETS OF EWSR1 FLII FUSION DN	Genes down-regulated in TC71 and EWS502 cells (Ewing's sarcoma) by EWSR1-FLI1 [GeneID=2130;2314] as inferred from RNAi knockdown of this fusion protein.	MSigDB	329	17	3.19E-14	2.03E-11
RIGGI EWING SARCOMA PROGENITOR UP	Genes up-regulated in mesenchymal stem cells (MSC) engineered to express EWS-FLI1 [GeneID=2130;2321] fusion protein.	MSigDB	430	18	1.98E-13	1.15E-10
WANG SMARCE1 TARGETS UP	Genes up-regulated in BT549 cells (breast cancer) by expression of SMARCE1 [GeneID=6605] off a retroviral vector.	MSigDB	280	15	6.47E-13	3.43E-10
HORIUCHI WTAP TARGETS UP	Genes up-regulated in primary endothelial cells (HUVEC) after knockdown of WTAP [GeneID=9589] by RNAi.	MSigDB	306	15	2.31E-12	1.13E-09
SENESE HDAC3 TARGETS UP	Genes up-regulated in U2OS cells (osteosarcoma) upon knockdown of HDAC3 [GeneID=8841] by RNAi.	MSigDB	501	18	2.56E-12	1.16E-09
MIYAGAWA TARGETS OF EWSR1 ETS FUSIONS UP	Genes commonly up-regulated in UET-13 cells (mesenchymal progenitor) by expression of EWSR1 [GeneID=2130] fusions with ETS transcription factors FLI1 and ERG [GeneID=2313 ,2078].	MSigDB	259	14	3.45E-12	1.46E-09
BENPORATH SUZ12 TARGETS	Set 'Suz12 targets': genes identified by ChIP on chip as targets of the Polycomb protein SUZ12 [GeneID=23512] in human embryonic stem cells.	MSigDB	1038	24	5.95E-12	2.37E-09
GRAESSMANN APOPTOSIS BY DOXORUBICIN_UP	Genes up-regulated in ME-A cells (breast cancer) undergoing apoptosis in response to doxorubicin [PubChem=31703].	MSigDB	1142	25	6.72E-12	2.52E-09
STK33 SKM UP	Genes up-regulated in SKM-1 cells (AML) after knockdown of STK33 [Gene ID=65975] by RNAi.	MSigDB	290	14	1.57E-11	5.54E-09
STK33 NOMO UP	Genes up-regulated in NOMO-1 cells (AML) after knockdown of STK33 [Gene ID=65975] by RNAi.	MSigDB	294	14	1.88E-11	6.30E-09
BENPORATH ES WITH H3K27ME3	Set 'H3K27 bound': genes posessing the trimethylated H3K27 (H3K27me3) mark in their promoters in human embryonic stem cells, as identified by ChIP on chip.	MSigDB	1118	24	2.74E-11	8.73E-09

Gene Set Name	Description	Source	Genes	Overlap	p-value	FDR
MANALO HYPOXIA UP	Genes up-regulated in response to both hypoxia and overexpression of an active form of HIF1A [GeneID=3091].	MSigDB	207	12	5.60E-11	1.70E-08
SWEET KRAS TARGETS UP	Genes upregulated in KRAS [GeneID=3845] knockdown vs control in a human cell line.	MSigDB	84	9	6.17E-11	1.79E-08
CSR LATE UP.V1 DN	Genes down-regulated in late serum response of CRL 2091 cells (foreskin fibroblasts).	MSigDB	170	11	1.11E-10	3.09E-08
ZWANG CLASS 3 TRANSIENTLY INDUCED BY EGF	Class III of genes transiently induced by EGF [GeneID =1950] in 184A1 cells (mammary epithelium).	MSigDB	222	12	1.26E-10	3.34E-08
HADDAD B LYMPHOCYTE PROGENITOR	Genes up-regulated in hematopoietic progenitor cells (HPC) of B lymphocyte lineage CD34+CD45RA+CD10+ [GeneID=947;5788;4311].	MSigDB	293	13	2.40E-10	5.89E-08
ZHANG TLX TARGETS 60HR UP	Genes up-regulated in neural stem cells (NSC) at 60 h after cre-lox knockout of TLX (NR2E1) [GeneID=7101].	MSigDB	293	13	2.40E-10	5.89E-08
MEISSNER BRAIN HCP WITH H3K4ME3 AND H3K27ME3	Genes with high-CpG-density promoters (HCP) bearing histone H3 dimethylation at K4 (H3K4me2) and trimethylation at K27 (H3K27me3) in brain.	MSigDB	1069	22	4.30E-10	1.01E-07
GOBERT OLIGODENDROCYTE DIFFERENTIATION_DN	Genes down-regulated during differentiation of Oli-Neu cells (oligodendroglial precursor) in response to PD174265 [PubChemID=4709].	MSigDB	1080	22	5.20E-10	1.18E-07
DAVICIONI TARGETS OF PAX FOXO1 FUSIONS UP	Genes up-regulated in RD cells (embryonal rhabdomyosarcoma, ERMS) by expression of PAX3- or PAX7-FOXO1 [GeneID=5077;5081;2308] fusions off retroviral vectors.	MSigDB	255	12	6.17E-10	1.35E-07
ZHOU INFLAMMATORY RESPONSE FIMA UP	Genes up-regulated in macrophages by P.gingivalis FimA pathogen.	MSigDB	544	16	8.11E-10	1.72E-07
MARSON BOUND BY FOXP3 UNSTIMULATED	Genes with promoters bound by FOXP3 [GeneID=50943] in unstimulated hybridoma cells.	MSigDB	1229	23	1.04E-09	2.13E-07
KOINUMA TARGETS OF SMAD2 OR SMAD3	Genes with promoters occupied by SMAD2 or SMAD3 [GeneID=4087, 4088] in HaCaT cells (keratinocyte) according to a ChIP-chip analysis.	MSigDB	824	19	1.14E-09	2.27E-07
CUI TCF21 TARGETS 2 DN	All significantly down-regulated genes in kidney glomeruli isolated from TCF21 [Gene ID=6943] knockout mice.	MSigDB	830	19	1.28E-09	2.48E-07
LEE BMP2 TARGETS UP	Genes up-regulated in uterus upon knockout of BMP2 [GeneID=650].	MSigDB	745	18	1.54E-09	2.89E-07
BENPORATH EED TARGETS	Set 'Eed targets': genes identified by ChIP on chip as targets of the Polycomb protein EED [GeneID=8726] in human embryonic stem cells.	MSigDB	1062	21	2.23E-09	4.06E-07
IVANOVA HEMATOPOIESIS STEM CELL AND PROGENITOR	Genes in the expression cluster 'HSC and Progenitors Shared': up-regulated in hematopoietic stem cells (HSC) and progenitors from adult bone marrow and fetal liver.	MSigDB	681	17	2.77E-09	4.87E-07
FEVR CTNNB1 TARGETS UP	Genes up-regulated in intestinal crypt cells upon deletion of CTNNB1 [GeneID=1499].	MSigDB	682	17	2.83E-09	4.87E-07
ELVIDGE HYPOXIA BY DMOG UP	Genes up-regulated in MCF7 cells (breast cancer) treated with hypoxia mimetic DMOG [PubChem=3080614].	MSigDB	130	9	3.15E-09	5.28E-07
HAN SATB1 TARGETS DN	Genes down-regulated in MDA-MB-231 cells (breast cancer) after knockdown of SATB1 [GeneID=6304] by RNAi.	MSigDB	442	14	3.77E-09	6.16E-07
GRAESSMANN RESPONSE TO MC AND DOXORUBICIN UP	Genes up-regulated in ME-A cells (breast cancer, sensitive to apoptotic stimuli) exposed to doxorubicin [PubChem=31703] in the presence of medium concentrate (MC) from ME-C cells (breast cancer, resistant to apoptotic stimuli).	MSigDB	612	16	4.33E-09	6.89E-07

Gene Set Name	Description	Source	Genes	Overlap	p-value	FDR
PLASARI TGFB1 TARGETS 1HR_UP	Genes up-regulated in MEF cells (embryonic fibroblast) upon stimulation with TGFB1 [GeneID=7040] for 1 h.	MSigDB	34	6	4.65E-09	7.22E-07
KONDO EZH2 TARGETS	Genes up-regulated in PC3 cells (prostate cancer) after EZH2 [GeneID=2146] knockdown by RNAi.	MSigDB	245	11	5.31E-09	7.92E-07
BAKKER FOXO3 TARGETS UP	Genes up-regulated in I/11 erythroblast cells upon expression of an activated form of FOXO3 [GeneID=2309].	MSigDB	61	7	5.35E-09	7.92E-07
PHOSPHORYLATION	Genes annotated by the GO term GO:0016310. The process of introducing a phosphate group into a molecule, usually with the formation of a phosphoric ester, a phosphoric anhydride or a phosphoric amide.	MSigDB	313	12	6.20E-09	8.97E-07
PASINI SUZ12 TARGETS DN	Genes down-regulated in ES (embryonic stem cells) with defficient SUZ12 [GeneID=23512].	MSigDB	315	12	6.65E-09	9.42E-07
BENPORATH PRC2 TARGETS	Set 'PRC2 targets': Polycomb Repression Complex 2 (PRC) targets; identified by ChIP on chip on human embryonic stem cells as genes that: posess the trimethylated H3K27 mark in their promoters and are bound by SUZ12 [GeneID=23512] and EED [GeneID=8726] Polycomb proteins.	MSigDB	652	16	1.05E-08	1.43E-06
SIGNAL TRANSDUCTION	Genes annotated by the GO term GO:0007165. The cascade of processes by which a signal interacts with a receptor, causing a change in the level or activity of a second messenger or other downstream target, and ultimately effecting a change in the functioning of the cell.	MSigDB	1634	25	1.06E-08	1.43E-06
PEDERSEN METASTASIS BY ERBB2 ISOFORM_7	Genes regulated in MCF7 cells (breast cancer) by expression of the truncated (611-CTF) form of ERBB2 [GeneID=2064] at 60 h time point.	MSigDB	403	13	1.12E-08	1.48E-06
KRIGE RESPONSE TO TOSEDOSTAT 6HR UP	Genes up-regulated in HL-60 cells (acute promyelocytic leukemia, APL) after treatment with the aminopeptidase inhibitor tosedostat (CHR-2797) [PubChem=15547703] for 6 h.	MSigDB	953	19	1.19E-08	1.55E-06
MASSARWEH TAMOXIFEN RESISTANCE UP	Genes up-regulated in breast cancer tumors (formed by MCF-7 xenografts) resistant to tamoxifen [PubChem=5376].	MSigDB	578	15	1.48E-08	1.89E-06
GRAESSMANN RESPONSE TO MC AND SERUM DEPRIVATION UP	Genes up-regulated in ME-A cells (breast cancer, sensitive to apoptotic stimuli) upon serum deprivation for 22 hr in the presence of medium concentrate (MC) from ME-C cells (breast cancer, resistant to apoptotic stimuli).	MSigDB	211	10	1.60E-08	2.00E-06
NUYTTEN NIPP1 TARGETS UP	Genes up-regulated in PC3 cells (prostate cancer) after knockdown of NIPP1 [GeneID=5511] by RNAi.	MSigDB	769	17	1.65E-08	2.02E-06
KRIGE RESPONSE TO TOSEDOSTAT 24HR UP	Genes up-regulated in HL-60 cells (acute promyelocytic leukemia, APL) after treatment with the aminopeptidase inhibitor tosedostat (CHR-2797) [PubChem=15547703] for 24 h.	MSigDB	783	17	2.14E-08	2.58E-06
SCHAEFFER PROSTATE DEVELOPMENT 48HR DN	Genes down-regulated in the urogenital sinus (UGS) of day E16 females exposed to the androgen dihydrotestosterone [PubChem=10635] for 48 h.	MSigDB	428	13	2.26E-08	2.67E-06
MITSIADES RESPONSE TO APLIDIN UP	Genes up-regulated in the MM1S cells (multiple myeloma) after treatment with aplidin [PubChem=44152164], a marine-derived compound with potential anti-cancer properties.	MSigDB	439	13	3.05E-08	3.53E-06
ELVIDGE HYPOXIA UP	Genes up-regulated in MCF7 cells (breast cancer) under hypoxia conditions.	MSigDB	171	9	3.46E-08	3.89E-06
MIYAGAWA TARGETS OF EWSR1 ETS FUSIONS DN	Genes commonly down-regulated in UET-13 cells (mesenchymal progenitor) by expression of EWSR1 [GeneID=2130] fusions with ETS transcription factors FLI1 and ERG [GeneID=2313 ,2078].	MSigDB	229	10	3.48E-08	3.89E-06
WONG ADULT TISSUE STEM MODULE	The 'adult tissue stem' module: genes coordinately up-regulated in a compendium of adult tissue stem cells.	MSigDB	721	16	4.21E-08	4.62E-06
VERHAAK GLIOBLASTOMA PRONEURAL	Genes correlated with proneural type of glioblastoma multiforme tumors.	MSigDB	177	9	4.66E-08	5.03E-06
RICKMAN TUMOR DIFFERENTIATED WELL VS POORLY DN	Down-regulated genes that vary between HNSCC (head and neck squamous cell carcinoma) groups formed on the basis of their level of pathological differentiation: well vs poorly differentiated tumors.	MSigDB	382	12	5.54E-08	5.88E-06

Gene Set Name	Description	Source	Genes	Overlap	p-value	FDR
GRAESSMANN APOPTOSIS BY SERUM DEPRIVATION UP	Genes up-regulated in ME-A cells (breast cancer) undergoing apoptosis upon serum starvation (5% to 0% FCS) for 22 hr.	MSigDB	552	14	6.04E-08	6.30E-06
CHYLA CBFA2T3 TARGETS UP	Genes up-regulated in immature bone marrow progenitor cells upon knock out of CBFA2T3 [GeneID=863].	MSigDB	387	12	6.38E-08	6.56E-06
SMID BREAST CANCER BASAL UP	Genes up-regulated in basal subtype of breast cancer samles.	MSigDB	648	15	6.58E-08	6.65E-06
GAUSSMANN MLL AF4 FUSION TARGETS F UP	Up-regualted genes from the set F (Fig. 5a): specific signature shared by cells expressing AF4-MLL [GeneID=4299;4297] alone and those expressing both AF4-MLL and MLL-AF4 fusion proteins.	MSigDB	185	9	6.81E-08	6.78E-06
CREIGHTON ENDOCRINE THERAPY RESISTANCE 5	The 'group 5 set' of genes associated with acquired endocrine therapy resistance in breast tumors expressing ESR1 but not ERBB2 [GeneID=2099;2064].	MSigDB	482	13	8.99E-08	8.80E-06
COULOUARN TEMPORAL TGFB1 SIGNATURE DN	'Early-TGFB1 signature': genes overexpressed in primary hepatocytes at an early phase of TGFB1 [GeneID=7040] treatment; is associated with a less invasive phenotype.	MSigDB	138	8	9.65E-08	9.31E-06
ALCALAY AML BY NPM1 LOCALIZATION UP	Genes up-regulated in acute myeloid leukemia (AML) with respect to cellular localization of NPM1 [GeneID=4869]: cytoplasmic vs. nucleolar.	MSigDB	140	8	1.08E-07	1.03E-05
GROSS HYPOXIA VIA ELK3 AND HIF1A UP	Genes up-regulated in SEND cells (skin endothelium) at hypoxia after knockdown of ELK3 [GeneID=2004] and HIF1A [GeneID=3091] by RNAi.	MSigDB	142	8	1.20E-07	1.13E-05
DURCHDEWALD SKIN CARCINOGENESIS DN	Genes down-regulated upon skin specific knockout of FOS [GeneID=2353] by cre-lox in the K5-SOS-F mice (express a constitutively active form of SOS1 [GeneID=6654] in the skin).	MSigDB	264	10	1.31E-07	1.21E-05
BMI1 DN MEL18 DN.V1 UP	Genes up-regulated in DAOY cells (medulloblastoma) upon knockdown of BMI1 and PCGF2 [Gene ID=648, 7703] genes by RNAi.	MSigDB	145	8	1.41E-07	1.29E-05
PODAR RESPONSE TO ADAPHOSTIN UP	Up-regulated genes in MM1.S cells (multiple myeloma) treated with adaphostin [PubChem=387042], a tyrosine kinase inhibitor with anticancer properties.	MSigDB	147	8	1.57E-07	1.41E-05
BOQUEST STEM CELL CULTURED VS FRESH UP	Genes up-regulated in cultured stromal stem cells from adipose tissue, compared to the freshly isolated cells.	MSigDB	425	12	1.75E-07	1.55E-05
CHANG CORE SERUM RESPONSE DN	Down-regulated genes in the canonical gene expression signature of the fibroblast core serum response (CSR) defined by the Stanford group.	MSigDB	209	9	1.92E-07	1.68E-05
NABA MATRISOME	Ensemble of genes encoding extracellular matrix and extracellular matrix-associated proteins	MSigDB	1028	18	2.02E-07	1.74E-05
SCHUETZ BREAST CANCER DUCTAL INVASIVE_UP	Genes up-regulated in invasive ductal carcinoma (IDC) relative to ductal carcinoma in situ (DCIS, non-invasive).	MSigDB	351	11	2.06E-07	1.75E-05
MIKKELSEN MCV6 HCP WITH H3K27ME3	Genes with high-CpG-density promoters (HCP) bearing the tri-methylation mark at H3K27 (H3K27me3) in MCV6 cells (embryonic fibroblasts trapped in a differentiated state).	MSigDB	435	12	2.24E-07	1.85E-05
SWEET LUNG CANCER KRAS DN	Genes down-regulated in the Kras2LA mouse lung cancer model with mutated KRAS [GeneID=3845].	MSigDB	435	12	2.24E-07	1.85E-05
SCHOEN NFKB SIGNALING	Genes down-regulated in A375 cells (melanoma) treated with KINK-1, a small molecule inhibitor of NFKB.	MSigDB	34	5	2.47E-07	2.01E-05
AMIT SERUM RESPONSE 120 MCF10A	Genes whose expression peaked at 120 min after stimulation of MCF10A cells with serum.	MSigDB	65	6	2.58E-07	2.08E-05
PROTEIN MODIFICATION PROCESS	Genes annotated by the GO term GO:0006464. The covalent alteration of one or more amino acids occurring in proteins, peptides and nascent polypeptides (co-translational, post-translational modifications). Includes the modification of charged tRNAs that are destined to occur in a protein (pre-translation modification).	MSigDB	631	14	3.05E-07	2.42E-05

Gene Set Name	Description	Source	Genes	Overlap	p-value	FDR
RODRIGUES THYROID CARCINOMA ANAPLASTIC DN	Genes down-regulated in anaplastic thyroid carcinoma (ATC) compared to normal thyroid tissue.	MSigDB	537	13	3.08E-07	2.42E-05
NAKAMURA TUMOR ZONE PERIPHERAL VS CENTRAL DN	Down-regulated genes in peripheral zone of human pancreatic cancer growing in the pancreas of nude mice compared to that of the tumor from the central zone.	MSigDB	634	14	3.23E-07	2.51E-05
SUNG METASTASIS STROMA UP	Genes up-regulated in metastatic vs non-metastatic stromal cells originated from either bone or prostate tissues.	MSigDB	110	7	3.31E-07	2.52E-05
CHEN HOXA5 TARGETS 9HR UP	Genes up-regulated 9 h after induction of HoxA5 [GeneID=3205] expression in a breast cancer cell line.	MSigDB	223	9	3.32E-07	2.52E-05
MONNIER POSTRADIATION TUMOR ESCAPE DN	The postradiation tumor escape signature: genes down-regulated in tumors from irradiated stroma vs those from non-irradiated stroma.	MSigDB	373	11	3.76E-07	2.79E-05
BROWN MYELOID CELL DEVELOPMENT UP	Genes defining differentiation potential of the bipotential myeloid cell line FDB.	MSigDB	165	8	3.81E-07	2.79E-05
UZONYI RESPONSE TO LEUKOTRIENE AND THROMBIN	Genes up-regulated in HUVEC cells (primary endothelium) after stimulation with leukotriene LTD4 [PubChem=3908] or thrombin (F2) [GeneID=2147] for 1 h.	MSigDB	37	5	3.83E-07	2.79E-05
MARTORIATI MDM4 TARGETS FETAL LIVER UP	Genes up-regulated in non-apoptotic tissues (fetal liver) after MDM4 [GeneID=4194] knockout.	MSigDB	227	9	3.86E-07	2.79E-05
DACOSTA UV RESPONSE VIA ERCC3 DN	Genes down-regulated in fibroblasts expressing mutant forms of ERCC3 [GeneID=2071] after UV irradiation.	MSigDB	855	16	4.16E-07	2.97E-05
MARTENS TRETINOIN RESPONSE UP	Genes up-regulated in NB4 cells (acute promyelocytic leukemia, APL) in response to tretinoin [PubChem=444795]; based on Chip-seq data.	MSigDB	857	16	4.29E-07	3.03E-05
BIOPOLYMER MODIFICATION	Genes annotated by the GO term GO:0043412. The covalent alteration of one or more monomeric units in a polypeptide, polynucleotide, polysaccharide, or other biological polymer, resulting in a change in its properties.	MSigDB	650	14	4.34E-07	3.04E-05
CHARAFE BREAST CANCER LUMINAL VS BASAL UP	Genes up-regulated in luminal-like breast cancer cell lines compared to the basal-like ones.	MSigDB	380	11	4.52E-07	3.13E-05
BASAKI YBX1 TARGETS DN	Genes down-regulated in SKOC-3 cells (ovarian cancer) after YB-1 (YBX1) [GeneID=4904] knockdown by RNAi.	MSigDB	384	11	5.01E-07	3.41E-05
KOKKINAKIS METHIONINE DEPRIVATION 96HR UP	Genes up-regulated in MEWO cells (melanoma) after 96 h of methionine [PubChem=876] deprivation.	MSigDB	117	7	5.03E-07	3.41E-05
CHICAS RB1 TARGETS CONFLUENT	Genes up-regulated in confluent IMR90 cells (fibroblast) after knockdown of RB1 [GeneID=5925] by RNAi.	MSigDB	567	13	5.67E-07	3.80E-05
POST TRANSLATIONAL PROTEIN MODIFICATION	Genes annotated by the GO term GO:0043687. The covalent alteration of one or more amino acids occurring in a protein after the protein has been completely translated and released from the ribosome.	MSigDB	476	12	5.81E-07	3.85E-05
CHIANG LIVER CANCER SUBCLASS CTNNB1 UP	Top 200 marker genes up-regulated in the 'CTNNB1' subclass of hepatocellular carcinoma (HCC); characterized by activated CTNNB1 [GeneID=1499].	MSigDB	176	8	6.21E-07	4.04E-05
NAGASHIMA NRG1 SIGNALING UP	Genes up-regulated in MCF7 cells (breast cancer) after stimulation with NRG1 [GeneID=3084].	MSigDB	176	8	6.21E-07	4.04E-05
AZARE NEOPLASTIC TRANSFORMATION BY STAT3 UP	Genes up-regulated in RWPE-1 cells (prostate cancer) upon expression of constitutively active form of STAT3 [GeneID=6774].	MSigDB	121	7	6.32E-07	4.07E-05
HOOI ST7 TARGETS DN	Genes down-regulated in PC-3 cells (prostate cancer) stably expressing ST7 [GeneID=7982] off a plasmid vector.	MSigDB	123	7	7.06E-07	4.50E-05

Supplementary Table 3. The list of GSEA gene signatures enriched in the 185 genes from supplementary table 2. The list was manually annotated for different categories as group, subgroup, and specifics. *P*-value was obtained from Hypergeometric test.

SETD1A-induced miRNAs that target the 31 genes upregulated in both SETD1A depleted A549 and MDA-MB-231 cells

miRNA	GeneID	GeneName
hsa-miR-106a	NM_004420	DUSP8
	NM_012326	MAPRE3
	NM_033285	TP53INP1
hsa-miR-125b	NM_004205	USP2
hsa-miR-140	NM_001106	ACVR2B
hsa-miR-142	NM_001186	BACH1
	NM_004277	SLC25A27
	NM_033285	TP53INP1
hsa-miR-15a	NM_020433	JPH2
hsa-miR-15b	NM_173582	PGM2L1
hsa-miR-186	NM_006763	BTG2
	NM_003275	TMOD1
	NM_018977	NLGN3
hsa-miR-199a	NM_015831	ACHE
	NM_015023	WDTC1
	NM_014731	ProSAPiP1
hsa-miR-204	NM_004694	SLC16A6
hsa-miR-20b	NM_006763	BTG2
hsa-miR-222	NM_001003940	BMF
	NM_001024401	SBK1
hsa-miR-24	NM_004420	DUSP8
	NM_015023	WDTC1
	NM_014454	SESN1
	NM_004205	USP2
hsa-miR-25	NM_005399	PRKAB2
hsa-miR-27a	NM_006763	BTG2
	NM_173039	AQP11
	NM_183376	ARRDC4
	NM_152271	LONRF1
	NM_014731	ProSAPiP1
	NM_173582	PGM2L1
	NM_031459	SESN2

miRNA	GeneID	GeneName
hsa-miR-296	NM_004714	DYRK1B
hsa-miR-29b	NM_014272	ADAMTS7
	NM_001003940	BMF
hsa-miR-30a	NM_005224	ARID3A
	NM_153690	FAM43A
	NM_033285	TP53INP1
hsa-miR-301a	NM_152271	LONRF1
hsa-miR-324	NM_015023	WDTC1
hsa-miR-32	NM_006763	BTG2
hsa-miR-328	NM_033285	TP53INP1
	NM_015023	WDTC1
hsa-miR-34a	NM_014731	ProSAPiP1
hsa-miR-424	NM_004714	DYRK1B
hsa-miR-452	NM_001935	DPP4
hsa-miR-532	NM_014731	ProSAPiP1
hsa-miR-545	NM_004420	DUSP8
hsa-miR-589	NM_014731	ProSAPiP1
hsa-miR-590-5p	NM_015831	ACHE
	NM_024607	PPP1R3B
	NM_005667	RNF103
	NM_006763	BTG2
	NM_033285	TP53INP1
hsa-miR-93	NM_031459	SESN2
hsa-miR-942	NM_001935	DPP4

Supplementary Table 4. List of SETD1A-regulated miRNAs that target the subset of 31 genes upregulated in SETD1A depleted MDA-MB-231 and A549 cells.

		BTG2 genomic sequence
P1	Forward	5’-GGCTCCTGTCCCATTCACT-3’
	Reverse	5’-TGTAGATTTTTCTGTCCCACACC-3’
P2	Forward	5’-CTGGTGTGGGACAGAAAAATC-3’
	Reverse	5’-GGGTTGTTCAGCACCTAAATG-3’
P3	Forward	5’-CACATTTAGGTGCTGAACAACC-3’
	Reverse	5’-CTGCTCACTGAGGGGAAAGA-3’
P4	Forward	5’-GGTCAGCCCTGTTTCCTGTA-3’
	Reverse	5’-CATATCTCTTAGATGGCCTCTTTCA-3’
P5	Forward	5’-ATCCAGCATTTGGGGAGAAG-3’
	Reverse	5’-GCTCACATGTCCCACATTTC-3’
P6	Forward	5’-CATGTGAGCATAGTAACCTGTTTTC-3’
	Reverse	5’-CCTGCCTCAATCTCTCACACT-3’
P7	Forward	5’-CCTTCAGTAGTGTGAGAGATTGAGG-3’
	Reverse	5’-CAGCAAATCTACAGCCTGGAT-3’
P8	Forward	5’-TTTGCTGATACTTAGAGGGCAGT-3’
	Reverse	5’-CATTCCCTAGCTCTTCTCTTGC-3’
P9	Forward	5’-GAGCAAGAGAAGAGCTAGGGAAT-3’
	Reverse	5’-CCAGTCACTCCGTCCAGAA-3’
P10	Forward	5’-GACCTTTCTGGACGGAGTGA-3’
	Reverse	5’-CAGTGGCTTTTCTCATCTGC-3’
P11	Forward	5’-TGCAGATGAGAAAAGCCACT-3’
	Reverse	5’-AGCCGTGTCCCTGTCTTTTT-3’
P12	Forward	5’-AAGACAGGGACACGGCTTC-3’
	Reverse	5’-CATCACAGGCACTCGGTAGA-3’
P13	Forward	5’-AGGCTTCACGGTGTTGGGTA-3’
	Reverse	5’-AGCCGTCTTCCTTCCTCATT-3’
P14	Forward	5’-CTTCACGGTGTTGGGTAGAAG-3’
	Reverse	5’-CGTATTTTTCCGTCCACTCC-3’
P15	Forward	5’-GCACTTAAGGAAAGTGAGAACTAGA-3’
	Reverse	5’-GGAAAGGGAGGCTGTCTTCT-3’
P16	Forward	5’-TTCCAAAGAGCCAAAAGGAA-3’
	Reverse	5’-GGCTCGAGTTAGAGACCACCT-3’
P17	Forward	5’-GGAGGAGGTGGCCTCTAACT-3’
	Reverse	5’-GCTGTCGTCAGTGCTAGGAA-3’
P18	Forward	5’-GGCGCAGGTTCTTAGCACT-3’
	Reverse	5’-GGCAGCGTTTTTCAGGAG-3’
P19	Forward	5’-AGGGTAACGCTGTTTTGTGG-3’
	Reverse	5’-TGAAGACCTTAAGCCTCTGCTC-3’
P20	Forward	5’-AGCGAGCAGAGGCTTAAGGT-3’
	Reverse	5’-CTGCCGCAGGAGTAGAAGAA-3’
P21	Forward	5’-CAGGGCCGTCTTTCTTCTAC-3’
	Reverse	5’-GAGGAGGACCCAGGAAACTG-3’
P22	Forward	5’-CAGTTTCCTGGGTCCTCCTC-3’
	Reverse	5’-CGTAATGAGACAGGGGCATAA-3’
P23	Forward	5’-GCTGGACCCTCGAGATCTTA-3’
	Reverse	5’-GAAGCCAGTCAGCAACCAGT-3’
P24	Forward	5’-CTGTGCCTGGGGTAGTCCAC-3’
	Reverse	5’-CGGGCTGCTTATCTCTTCAC-3’
P25	Forward	5’-CCTTCAAGTTGGGAGGTGAA-3’
	Reverse	5’-TCGGAGAACCTTCCTTAGCA-3’
P26	Forward	5’-TCACTCCCTTAGGCACTGCT-3’
	Reverse	5’-GAAGGGTGTGACTGGGACTG-3’
P27	Forward	5’-CAGTCCCAGTCACACCCTTC-3’
	Reverse	5’-TTCCTGCTTCCAGGACAAGT-3’
P28	Forward	5’-CATACACTTGTCCTGGAAGCAG-3’
	Reverse	5’-GTGGAGGGTCAAGGGACAG-3’
P29	Forward	5’-ACCCCGCCCTATGGTAGTAT-3’
	Reverse	5’-GGGTCCATCTTGTGGTTGAT-3’
P30	Forward	5’-GCATTCGCATCAACCACA-3’
	Reverse	5’-GGGCCTCCTCGTACAAGAC-3’
P31	Forward	5’-CTCCATCTGCGTCTTGTACG-3’
	Reverse	5’-GAGGTATGTGGTGGCCTGTT-3’

		miR-32 genomic sequence
P1	Forward	5’-TGAGACAGAGTTTTGCTCTTGC -3’
	Reverse	5’-GCCAAGTGTGGTGTGGTAC -3’
P2	Forward	5’-GCAGGACGAAATTGCCTAAT -3’
	Reverse	5’-GGCAGCAAGAGCAAAACTCT -3’
		**miR-590 genomic sequence**
P1	Forward	5’-TTAGAGCCAACCAGCAGCTC-3’
	Reverse	5’-ACTGCAGTTCCCACCAGAAC-3’
		**TMEM245 genomic sequence**
P1	Forward	5’-GCCAGAATCCCTGAGTTTGA-3’
	Reverse	5’-GGCCCTATTCTAAGTGGCTCA-3’
P2	Forward	5’-GGAGTGAGCCACTTAGAATAGGG-3’
	Reverse	5’-AGGCGCCACATTCAGATATT-3’
P3	Forward	5’-TATCTGAATGTGGCGCCTTT-3’
	Reverse	5’-AAGCGCTTGCAGGAAACTAC-3’
P4	Forward	5’-CGCTTGGTGGGTCTGTAAAC-3’
	Reverse	5’-TAATGGGAGTCGGGCTAGAA-3’
P5	Forward	5’-TAGCCCGACTCCCATTACC-3’
	Reverse	5’-GCCTGCTTAATGGGCTTGT-3’
P6	Forward	5’-TTAAGCAGGCCTTCTACAACA-3’
	Reverse	5’-AGCTCTTGAAGGGGTGCAG-3’
P7	Forward	5’-GCACTTTTCTGCACCCCTTC-3’
	Reverse	5’-CCGTAGTCGACGAAGCAGAG-3’
P8	Forward	5’-TCCTGTACGGCCTCTACTGC-3’
	Reverse	5’-GAGGAACAGCTGCAGGATTG-3’
P9	Forward	5’-CAATCCTGCAGCTGTTCCTC-3’
	Reverse	5’-GGGCCTATTAGGATTGAAACAA-3’
P10	Forward	5’-TTTTCGCCTTGTTTCAATCC-3’
	Reverse	5’-CCCTTCCAGCTCTGACATTC-3
		**EIF4H genomic sequence**
P1	Forward	5’-TGCCTGTAATCCCAGCACTT-3’
	Reverse	5’-TAACTGGGGTGACAGACGTG-3’
P2	Forward	5’-TCTTTTTCCAAGGCAACGAC-3’
	Reverse	5’-GTGGGATGCAGACACGAAT-3’
P3	Forward	5’-CTGCGTCCAAGGGATTTG-3’
	Reverse	5’-CGATCGTCGTAGGTGTCGAA-3’
P4	Forward	5’-TATATTGCGGGGTCCTTCCT-3’
	Reverse	5’-CGTCGTAGGTGTCGAAGTCC-3’

Supplementary Table 5. Sequences of primers used for qChIP analysis of the genomic regions of interest.

Symbol		target sequence	Symbol		target sequence
DOT1	Forward	5'-GCTGCCGGTCTACGATAAACA-3'	PRDM2	Forward	5'-ATTTGGGATGGATGTGCATT-3'
	Reverse	5'-AGCTTGAGATCCGGGATTTCT-3'		Reverse	5'-CAGCCAGTTTCCCTTCTCTG-3'
Ezh1	Forward	5'-CCCTGACCTCTGTCTTACTTGTGGA-3'	PRDM5	Forward	5'-TACGTGCCGGACAGGTTCT-3'
	Reverse	5'-ACGTCAGATGGTGCCAGCAATA-3'		Reverse	5'-TTCACCCTTTCGCACTCTGC-3'
Ezh2	Forward	5'-AAGTACACGGGGATAGAGAATGT-3'	PRDM7	Forward	5'-GAACCAAAGCCAGAGATCCA-3'
	Reverse	5'-GGTGGGCGGCTTTCTTTATCA-3'		Reverse	5'-GAGAGGAGTGATTGCGTTCC-3'
EHMT2	Forward	5'-CCGAGAGAGTTCATGGCTCTT-3'	PRDM8	Forward	5'-ATATCTTTCGGGTAGACACCTCA-3'
	Reverse	5'-TGGGCAGGGTTTCTTCACTAC-3'		Reverse	5'-CTGGCCGATTGGACCAACC-3'
SUV39H1	Forward	5'-ATCCGCGAACAGGAATATTACC-3'	PRDM9	Forward	5'-TTGGATGGCCTTAAGAGTGG-3'
	Reverse	5'-GAGGATACGCACACACTTGAGATT-3'		Reverse	5'-TCTGAGCCACTTGCATTCAG-3'
SUV39H2	Forward	5'-TCTATGACAACAAGGGAATCACG-3'	PRDM10	Forward	5'-GACACGGATCTGGATGACTGG-3'
	Reverse	5'-GAGACACATTGCCGTATCGAG-3'		Reverse	5'-GCGCGTTATTGCACTCCTC-3'
SUV420H1	Forward	5'-TCGAGAGGAGGTCGAACAGAT-3'	PRDM11	Forward	5'-TGGAGAGAGTGACGTGCGAT-3'
	Reverse	5'-AGAGGATGGTACATAGCGACTC-3'		Reverse	5'-ACTTATAGCGGTTGTTCTTGTCC-3'
SUV420H2	Forward	5'-AGTGGCTTTACCATCCTGCC-3'	PRDM12	Forward	5'-GGGAGGTGTTCAATGAGGATG-3'
	Reverse	5'-CACGAGTGGACACGATCTTG-3'		Reverse	5'-TCTGCTCCTGTTCGTTACGTG-3'
MLL	Forward	5'--CAGCCATTTGCTACGCTACC-3'	PRDM13	Forward	5'-GCAGACTTACCCGGAGGAC-3'
	Reverse	5'-TCAGTAAGAACTGGTGGATCAGG-3'		Reverse	5'-CTCGCAATGCTCGGTAGAAGA-3'
MLL2	Forward	5'-ACCTGGGAATGACTCTAAGATGT-3'	PRDM14	Forward	5'-GAGCCTTCAGGTCACAGAGC-3'
	Reverse	5'-CACGCCTTGCACTTCCAAGA-3'		Reverse	5'-ACTCTTCAGAGGGCCCAGAT-3'
MLL3	Forward	5'-GGACAGAGAAAAGAACGATCTCC-3'	PRDM16	Forward	5'-AGACTTCGGATGGGAGCAAAT-3'
	Reverse	5'-TGGGTGCTTACACTTACACAAGA-3'		Reverse	5'-TCCACGCAGAACTTCTCACTG-3'
MLL5	Forward	5'-TTGCCCTATGCGGACCATAAT-3'	SMYD1	Forward	5'-TCGGAGGTCCTTTCCTACCT-3'
	Reverse	5'-CCATCCTCAGATGTGCTGATTG-3'		Reverse	5'-TATAGCCGTCCACCATCCTC-3'
SETD1A	Forward	5'-GGCCAGATTCATCAACCACT-3'	SMYD2	Forward	5'-CTCCAAGCATCTCGGATTCCC-3'
	Reverse	5'-CGATCTTCTTCTGGGACTCG-3'		Reverse	5'-TGCAACATCAGGAAATATCGCTG-3'
SETD1B	Forward	5'-GCTGTCGGTGCCCAAATTC-3'	SMYD3	Forward	5'-AGTTGGTGTTGGCCTATATCCC-3'
	Reverse	5'-CTCAGGAAGTTTTCACGGATGTT-3'		Reverse	5'-ACACAATCGAACAGTTGGGGT-3'
SETDB1	Forward	5'-TAAGACTTGGCACAAAGGCAC-3'	SMYD4	Forward	5'-GGTGGGAAAGGACTCGGAC-3'
	Reverse	5'-CTGGCCCAACTGTCTGGATG-3'		Reverse	5'-GGTTAGCATGACACAGTGACAT-3'
SETDB2	Forward	5'-AGGGAGCACAAAAGGACTCA-3'	SMYD5	Forward	5'-AATGCACTTTATCGCTACCGAG-3'
	Reverse	5'-GTGGTCGCCTGGTTACATCT-3'		Reverse	5'-CTGCCAACCGACATTCTGC-3'
SETD2	Forward	5'-ACAGGTTTCATCAAAGGACCAAT-3'	SETMAR	Forward	5'-GAAGCGGCAAAGACGACAC-3'
	Reverse	5'-GGGCAAAAATCGACTAGAAGCA-3		Reverse	5'-GAGTGGGATCAATGTCTGCTC-3'
SETD6	Forward	5'-GGAGAGCTGTTGTTCGTGGT-3'	BTG2	Forward	5'-CAGAGCACTACAAACACCACTG-3'
	Reverse	5'-GAGCGCAAAGTAGGGCCTC-3'		Reverse	5'-CTGAGTCCGATCTGGCTGG-3'
SETD7	Forward	5'-GCACCCTGGAGGGGTATTAT-3'	ARID3A	Forward	5'-ACCACGGCGACTGGACTTA-3'
	Reverse	5'-ACATACGTGCCCTGGAGAAC-3'		Reverse	5'-CACAGGTGTCCCTCGCTTC-3'
SETD8	Forward	5'-ACCGACGGGGAGAACGTATT-3'	SESN1	Forward	5'-CTACATTGGAATAATGGCTGCGG-3'
	Reverse	5'-GCATTCCAGAGCATTTGTTCG-3'		Reverse	5'-AGGTCTATGGGCTAACACTTTGT-3'
NSD1	Forward	5'-AGAAAAAGTCTACGCCACTGAAG-3'	TP53INP1	Forward	5'-TTCCTCCAACCAAGAACCAGA-3'
	Reverse	5'-ATAGGGCCTCCGGTTGGAA-3'		Reverse	5'-GCTCAGTAGGTGACTCTTCACT-3'
WHSC1	Forward	5'-GAGAGCACGCTACAACACCA-3'	TMEM245	Forward	5'-TTCTGGGGCAGAGTTACCAG-3'
	Reverse	5'-GTGTGTCCTCAGCTTCAGCA-3'		Reverse	5'-GGCCAAGTTTGCCAGAGTAG-3'
WHSC1L1	Forward	5'-GGTGGGAACCTATCCTTGGT-3'	EIF4H	Forward	5'-ATGGCGGACTTCGACACCTA-3'
	Reverse	5'-CGGGCACCTCTTGTGTTAAT-3'		Reverse	5'-GCCCTGAACCGTATTGAAAGGTA-3'
ASH1L	Forward	5'-ACACTGTCCTTCAAAACGAGAC-3'	WDR82	Forward	5'-CAGATACACTCATGCAGCAAACA-3'
	Reverse	5'-GAAGAGTAGATGGCGTTGCATTA-3'		Reverse	5'-GTCATGCAAGGACAAGTAACGA-3'
EVI1	Forward	5'-GCGAAGACTATCCCCATGAAAC-3'	GAPDH	Forward	5'-AGTCCTTCCACGATACCAAAGT-3'
	Reverse	5'-GCAGCGATATTGCCGTTCT-3'		Reverse	5'-CATGAGAAGTATGACAACAGCCT-3'
PRDM1	Forward	5'-CTACCCTTATCCCGGAGAGC-3'	β-Actin	Forward	5'-CTCTTCCAGCCTTCCTTCCT-3'
	Reverse	5'-GCTCGGTTGCTTTAGACTGC-3'		Reverse	5'-AGCACTGTGTTGGCGTACAG-3'

Supplementary Table 6. Sequences of forward and reverse primers used for qPCR analysis of all the genes shown.

Supplementary References

1. Selamat SA, *et al*. Genome-scale analysis of DNA methylation in lung adenocarcinoma and integration with mRNA expression. *Genome Res* **22**, 1197-1211 (2012)

2. Tomlins SA, *et al*. Integrative molecular concept modeling of prostate cancer progression. *Nat Genet* **39**, 41-51 (2007)

3. Comprehensive molecular portraits of human breast tumours. *Nature* **490**, 61-70 (2012).

4. Chandran UR, *et al.* Gene expression profiles of prostate cancer reveal involvement of multiple molecular pathways in the metastatic process. *BMC Cancer* **7**, 64 (2007).

5. Taylor BS, *et al.* Integrative genomic profiling of human prostate cancer. *Cancer Cell* **18**, 11-22 (2010).

6. Perou, CM *et al*. Molecular portraits of human breast tumours. *Nature* **406**, 747-52 (2000)

7. Raponi N *et al*. Gene expression signatures for predicting prognosis of squamous cell and adenocarcinomas of the lung. *Cancer Res* **66**, 7466-72 (2006)

8. Zhu CQ *et al*. Prognostic and predictive gene signature for adjuvant chemotherapy in resected non-small-cell lung cancer. *J Clin Oncol* **28**, 4417-24 (2010)

9. Li JH, Liu S, Zhou H, Qu LH, Yang JH. starBase v2.0: decoding miRNA-ceRNA, miRNA-ncRNA and protein-RNA interaction networks from large-scale CLIP-Seq data. *Nucleic Acids Res* **42**, D92-97 (2014).

10. Yang JH, Li JH, Shao P, Zhou H, Chen YQ, Qu LH. starBase: a database for exploring microRNA-mRNA interaction maps from Argonaute CLIP-Seq and Degradome-Seq data. *Nucleic Acids Res* **39**, D202-209 (2011).

11. Monteys AM, *et al.* Structure and activity of putative intronic miRNA promoters. *RNA* **16**, 495-505 (2010).

12. Nakazato, T. *et al*. BioCompass: A novel functional inference tool that utilizes MeSH hierarchy to analyze groups of genes. *In Silico Biology*, **8**(1): 53-61 (2008)

13. Nakazato, T. *et al*. Gendoo: Functional profiling of gene and disease features

using MeSH vocabulary. *Nucleic Acids Research,* **37**(Suppl. 2) (Web Server Issue):W166-W169 (2009)
